# Evaluation on the tribological performance of ring/liner system under cylinder deactivation with consideration of cylinder liner deformation and oil supply

**DOI:** 10.1371/journal.pone.0204179

**Published:** 2018-09-17

**Authors:** Yanjun Lu, Cheng Liu, Yongfang Zhang, Jiahui Wang, Kangrui Yao, Yafeng Du, Norbert Müller

**Affiliations:** 1 School of Mechanical and Precision Instrument Engineering, Xi’an University of Technology, Xi’an, China; 2 State Key Laboratory of Digital Manufacturing Equipment and Technology, Huazhong University of Science and Technology, Wuhan, China; 3 School of Printing, Packaging Engineering and Digital Media Technology, Xi’an University of Technology, Xi’an, China; 4 Key Laboratory of Manufacturing Equipment of Shaanxi Province, Xi’an University of Technology, Xi’an, China; 5 College of Engineering, Michigan State University, East Lansing, Michigan, United States of America; Lanzhou University of Technology, CHINA

## Abstract

In gasoline engines, CDA (cylinder deactivation) affects greatly the tribological performance of ring/liner system while reducing the emissions and improving the fuel economy. The analyses on the tribological performance of ring/liner system under the CDA mainly focus on the ideal circular cylinder liner and fixed fully flooded lubrication condition. In this study, a numerical investigation on the tribological performance of a compression ring-cylinder liner system is presented under the CDA with consideration of the cylinder liner deformation and the transition between the fully flooded and starved lubrication conditions. A mixed lubrication model coupled with oil transport model and JFO (Jacobson-Floberg-Olsson) conservative cavitation algorithm is proposed to evaluate the frictional properties. Based on the model, the tribological performance is investigated under the standard operation condition and the CDA. Meanwhile, the influence of cylinder liner deformation and oil supply on the tribological performance is also evaluated. Results show that the tribological performance of the compression ring-cylinder liner system is greatly changed when the CDA is adopted. In particular, under the CDA, the overall power loss and FMEP (friction mean effective pressure) value are increased about 27.29% and 53.51%. The study also demonstrates the necessity to consider the cylinder liner deformation and oil supply in the simulation of compression ring-cylinder liner system under the CDA.

## Introduction

Compression ring-cylinder liner system in internal combustion engine usually acts as a seal between the combustion chamber and the crankcase [1–4]. In generally, the tribological properties of the compression ring-cylinder liner system affect greatly the seal performance and energy efficiency of the engine because the surface materials of cylinder liner and compression ring wear out during the operation [5, 6]. Under this case, the tribological properties of the compression ring-cylinder liner system have been paid special attention to improve the seal performance and energy efficiency maximally.

Jeng et al. [7, 8] proposed a one-dimensional lubrication model to investigate the tribological performance of the compression ring-cylinder liner system under both the fully flooded and starved lubrication conditions, and this model was also extended to predict the performance of the piston ring pack. Usman et al. [9] and Livanois et al. [10] evaluated the tribological performance of the compression ring-cylinder liner system for various oil viscosities, engine speeds, and loads. In their studies, significant influence of oil viscosity, engine speed, and load on the tribological performance was observed. In recently, for the purpose of improving the tribological performance of the compression ring-cylinder liner system, Zhang et al. [11] optimized the face shape of the compression ring by using the inverse method. Etsion and his research group [12, 13] textured micro dimples on the compression ring, and a parametric analysis was conducted to obtain the optimal dimple parameters for maximum friction reduction. In these works, a dominant hydrodynamic lubrication regime was considered. However, besides the hydrodynamic lubrication regime, a mixed lubrication regime is typically encountered at piston top and bottom dead centers [14, 15]. Hence, the asperity contact in the mixed lubrication regime needs to be further considered [16]. By considering the asperity contact behaviors in the mixed lubrication regime, Rahmani et al. [17] studied the influence of liner temperature on the frictional characteristics and engine emissions of the compression ring-cylinder liner system. Morris et al. [18] investigated the influence of the axial profiles and surface roughness of compression ring on the performance of the compression ring-cylinder liner system. The reasonable axial profile and surface roughness of compression ring were chosen to obtain superior frictional performance. Meng et al. [19] and Yin et al. [20] evaluated the frictional properties of the compression ring-cylinder liner system with dimple texturing under the mixed lubrication regime. A great improving of the mixed lubrication performance was observed for the compression ring-cylinder liner system with dimple texturing. However, the analyses in these works were mainly focused on the compression ring-cylinder liner system with ideal circular cylinder liner. In actuality, the cylinder liner is usually deformed because of the large load and inevitable manufacturing errors [21]. For the deformed cylinder liner, Dunaevsky et al. [22] provided a method to model the profile of the deformed cylinder liner based on the Fourier transform. Based on the method of Dunaevsky et al. [22], Hu et al. [23] and Mishra et al. [24] investigated the mixed lubrication performance of the compression ring-cylinder liner system with a deformed cylinder liner. Usman et al. [25] evaluated the sensitivity of mixed lubrication performance to the magnitude of cylinder liner deformation under warm-up engine condition. Their works indicated that the cylinder liner deformation affects greatly the frictional performance of the compression ring-cylinder liner system. However, a pure fully flooded lubrication condition was assumed in their works. Due to the scraping effect of the oil ring, insufficient amount of oil film will be transported/supplied to the compression ring-cylinder liner system, and the lubrication condition of compression ring-cylinder liner system will change from the fully flooded lubrication to starved lubrication when the compression ring reaches near the middle of the strokes [26]. Meng et al. [27] and Tian et al. [28] investigated the frictional performance of the compression ring-cylinder liner system with consideration of the starved lubrication condition in the engine cycle, and the start position of lubrication zone was determined by solving the flow conservation equations. In their work, the starved lubrication performance of the compression ring-cylinder liner system was analyzed with also focus on the ideal circular cylinder liner.

Moreover, the above-mentioned studies have been conducted for the conventional internal combustion engines under standard operation condition. However, in modern design of internal combustion engines, some advanced technologies, such as cylinder deactivation (CDA), variable valve actuation (VVA), turbo-charging system, and stop-start management [29, 30], have been widely adopted to reduce the emissions and improve the fuel economy [31]. In these technologies, the CDA is usually adopted in multi-cylinder engines, and can output a desired power by varying the number of active cylinders [32, 33]. Although the CDA shows potential for improving fuel economy, it also promotes certain undesired side-effects in some engine conjunctions because the working condition changes greatly (ex. cylinder pressure, cylinder liner temperature, and etc.) [34, 35]. In order to evaluate the side effects of the CDA on the tribological performance of the compression ring-cylinder liner system, a mixed lubrication model was proposed by Rahmani and his coworkers [34, 36], and the minimum oil film thickness, friction, and power loss were evaluated for the compression ring-cylinder liner system under the CDA. It was showed that the CDA affects greatly the frictional performance of the compression ring-cylinder liner system, and a significant increase in power loss was observed for the compression ring-cylinder liner system under the CDA. However, the fully flooded lubrication condition and ideal circular cylinder liner were assumed in their works.

On the basis of the aforementioned studies, the analyses on the tribological performance of the compression ring-cylinder liner system are mainly conducted for the conventional engines (i.e., no use of the CDA), or focused on the engines under the CDA with assumptions of ideal circular cylinder liner and fixed fully flooded lubrication condition. In this study, the tribological performance of the compression ring-cylinder liner system under the CDA is analyzed with consideration of the cylinder liner deformation and the transition between the fully flooded lubrication and starved lubrication. In the analysis, an oil transport model coupled with JFO (Jacobson-Floberg-Olsson) conservative cavitation algorithm is proposed to predict the transient lubrication condition (i.e., fully flooded/starved). A mixed lubrication model is employed to evaluate the frictional properties of the compression ring-cylinder liner system under the mixed lubrication regime. On this basis, the influence of cylinder liner deformation and oil supply on the frictional performance is evaluated, and the tribological performance of the compression ring-cylinder liner system is also compared between the CDA and standard operation condition.

## Method

### Governing equation

**Fig 1:**
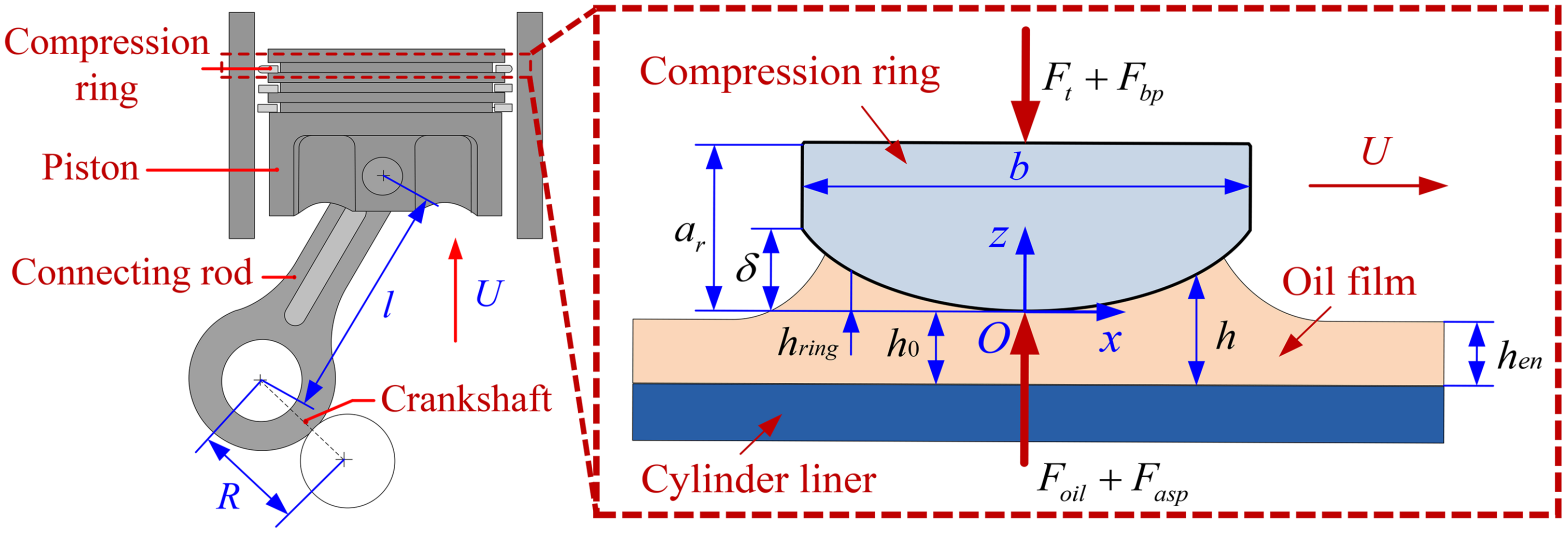
Schematic diagram of the compression ring-cylinder liner system.

Fig 1 shows the schematic diagram of the compression ring-cylinder liner system. In order to evaluate the tribological performance of the compression ring-cylinder liner system under the CDA accurately, the surface roughness and oil film cavitation phenomenon (i.e., rupture and reformulation of oil film) are considered, and an average Reynolds equation coupled with mass conservative JFO cavitation algorithm [37] is adopted to calculate the pressure distribution of the oil film. In the average Reynolds equation, the side-leakage Couette flow of the oil film in the circumferential direction is not considered [34]. This is justified by assuming that there is no relative motion in the circumferential direction. Therefore, the average Reynolds equation coupled with JFO cavitation algorithm can be written as [37]:

$$\frac{\partial }{\partial x}\left[\phi_{x}\frac{\rho h^{3}}{\mu }\frac{\partial p}{\partial x}\right]+\frac{\partial }{\partial y}\left[\phi_{y}\frac{\rho h^{3}}{\mu }\frac{\partial p}{\partial y}\right]=6U\phi_{c}\frac{\partial }{\partial x}(\theta_{c}\rho h)+6U\sigma \frac{\partial }{\partial x}(\theta_{c}\phi_{s}\rho )+12\phi_{c}\frac{\partial }{\partial t}(\theta_{c}\rho h)$$ (1)

where *x* and *y* are the axial and circumferential coordinates, *p* is the pressure of oil film, *μ* is the oil viscosity, *ρ* is the oil density, *h* is the oil film thickness, $\sigma =\sqrt{\sigma_{1}^{2}+\sigma_{2}^{2}}$ is the comprehensive surface roughness, *σ*_1_ is the surface roughness of compression ring, *σ*_2_ is the surface roughness of cylinder liner, *t* is the time, *θ*_*c*_ is the oil film saturation, *U* is the relative speed of compression ring [14]. *ϕ*_*x*_ and *ϕ*_*y*_ are the pressure flow factors, *ϕ*_*s*_ is the shear flow factor, *ϕ*_*c*_ is the contact factor. The flow factors defined by Patir et al. [38, 39] and Wu et al. [40] are used with an assumption of Gaussian distributed asperity heights, and their expressions are given in Appendix A of S1 File. It should be noted that the cylinder liner is cross-hatched/honed, and the surface asperity heights are usually not Gaussian distributed. However, according to the engine testing results of Gore et al. [41], after the initial running-in period, a plateau surface can be achieved, and the asperity heights of the cylinder liner surface are closely to a Gaussian distribution. In this study, the cylinder liner after its initial running-in period is considered. Therefore, the assumption of Gaussian distributed asperity heights of the cylinder liner surface is considered to be acceptable and reasonable [36, 42, 43, 44]. A calculation on the flow factors of non-Gaussian cylinder liner surface can refer to a study conducted by Leighton et al [45].

The oil film saturation *θ*_*c*_ is given as [46]:

$$\left\{ \begin{array}{l} \theta_{c}=1\;\text{if}\;p>p_{c}\;\;\;\;\text{full}\;\text{film}\;\text{region}\\ \theta_{c}<1\;\text{if}\;p=p_{c}\;\;\;\;\text{cavitation}\;\text{region} \end{array}\right.$$ (2)

where *p*_*c*_ is the cavitation pressure. It is worth noting that the cavitation region and cavitation boundary (i.e., rupture and reformulation boundaries) can be distinguished and addressed based on the value of saturation of oil film *θ*_*c*_ in the simulation. More detailed information about the mass conservative JFO cavitation algorithm can refer to the other representative works [46, 47].

### Expression of oil film thickness

For solving the average Reynolds equation, it is necessary to describe the oil film thickness between the compression ring and cylinder liner reasonably. In this study, a barrel-shaped compression ring is considered because it is most commonly used in the gasoline engines [11, 14, 27]. Meanwhile, according to a large number of research results [21–25], the cylinder liner in the fired engine condition is usually out-of-roundness because of the cylinder liner deformation caused by the thermal load, mechanical load, manufacturing error, and load difference between thrust and anti-thrust sides. Thus, the oil film thickness between the barrel-shaped compression ring and deformed cylinder liner can be expressed as:

$$h=h_{\text{0}}+h_{ring}+h_{liner}$$ (3)

where *h*_0_ is the minimum oil film thickness between the compression ring and cylinder liner, *h*_*ring*_ is the gap between the compression ring and cylinder liner caused by the axial profile of barrel-shaped compression ring, and it can be described by a parabolic equation [11]. *h*_*liner*_ is the gap between the compression ring and cylinder liner caused by the cylinder liner deformation and compression ring conformability. The expressions of *h*_*ring*_ and *h*_*liner*_ can be written as follows [11, 25, 27]:

$$h_{ring}\text{(}x\text{)}=4\frac{\delta x^{2}}{b^{2}}$$ (4)

$$h_{ring}=\Delta R-U_{n}$$ (5)

where *δ* is the crown height of compression ring, *b* is the axial width of compression ring. Δ*R* is the variation of cylinder liner radius from its inscribed circle, *U*_*n*_ is the elastic deformation of compression ring, and they will be discussed in the next section.

### Cylinder liner deformation and compression ring conformability

**Fig 2:**
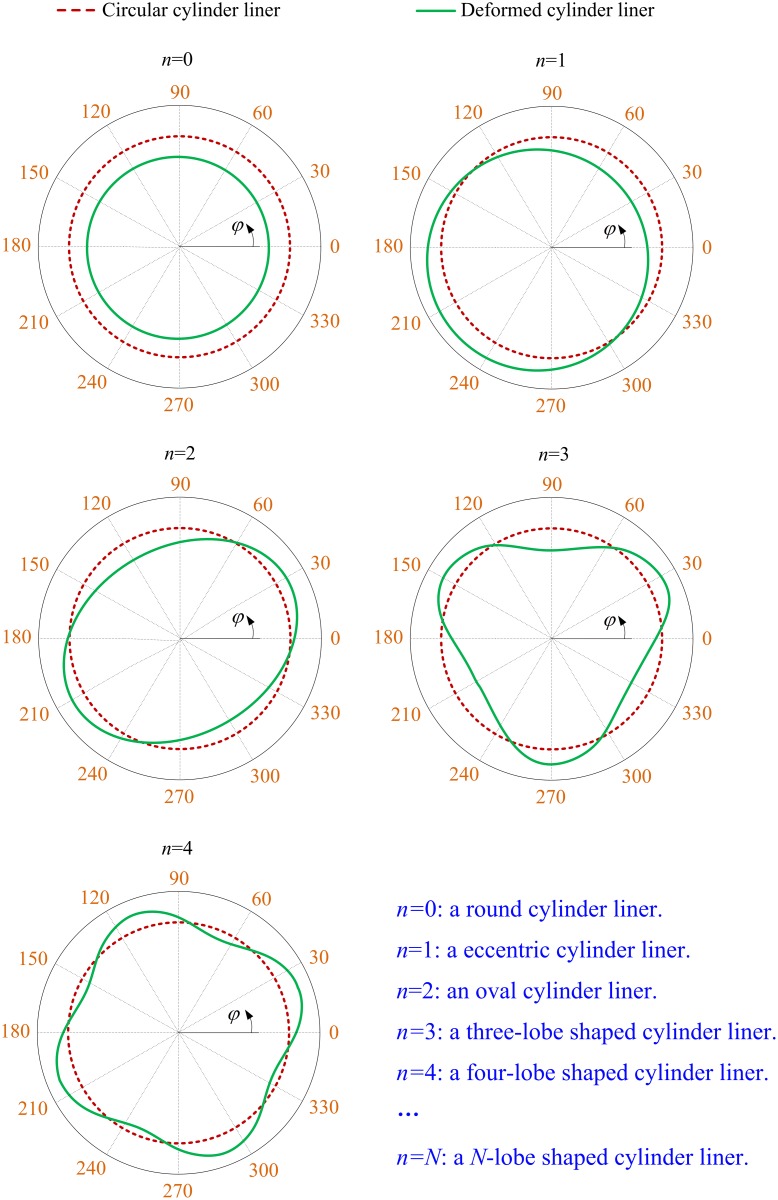
Types of cylinder liner deformation for the first five orders.

According to the work of Ma et al. [48], the cylinder liner deformation varies the radius of cylinder liner, and the variation of cylinder liner radius from its ideal circular shape Δ*R*_*cir*_ at an axial cross-section can be expressed by using a Fourier series.

$$\Delta R_{cir}=\sum_{n=0}^{N}\left[A_{n}\text{cos}(n\varphi )+B_{n}\text{sin}(n\varphi )\right]$$ (6)

where *N* is the maximum order of the Fourier series, *φ* is the circumferential position, *n* is the order of the Fourier series, *A*_*n*_ and *B*_*n*_ are the Fourier coefficients for the order *n*. The order *n* is defined as a non-negative integer. Different values of order *n* mean different types of cylinder liner deformation [22]. Generally, the cylinder liner deformation can be modeled accurately by using the Fourier series with first few orders. Fig 2 shows the types of cylinder liner deformation for the first five orders.

Under the working conditions, the deformed cylinder liner mainly exhibits an oval shape (i.e., *n* = 2) and a four-lobe shape (*n* = 4) [49]. However, as mentioned in the works of Rahmani et al. [50] and Usman et al [51], the compression ring also elastically deforms to accommodate the radial radius variation of cylinder liner when the cylinder liner is deformed, and the elastic deformation of compression ring can completely accommodate the radial radius variation of the deformed cylinder liner with *n* = 2 [52]. This means that the gap between the compression ring and cylinder liner caused by the cylinder liner deformation after considering the compression ring elastic deformation is mainly the outcome of the cylinder liner deformation with *n* = 4 [51]. Therefore, *n* = 4 is chosen to model the measured variation of cylinder liner radius from its ideal circular shape Δ*R*_*cir*_.

**Fig 3:**
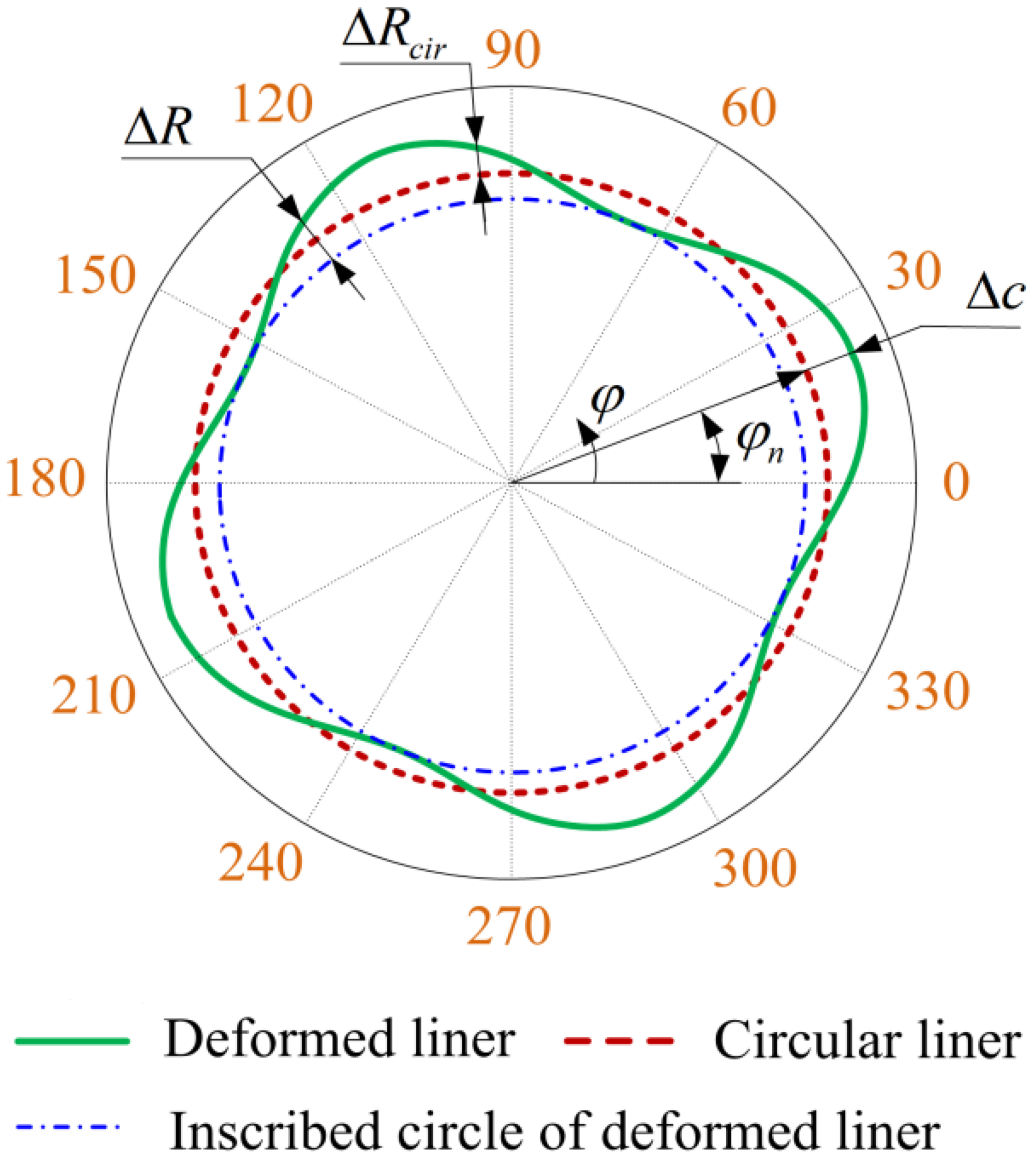
Schematic diagram of the deformed cylinder liner with *n* = 4.

In order to facilitate the calculation, the expression of Δ*R*_*cir*_ can be rewritten as [50]:

$$\Delta R_{cir}=\sum_{n=0}^{N}\left\{\Delta c\text{cos}\left[n(\varphi -\varphi_{n})\right]\right\}$$ (7)

with

$$\{\begin{array}{l} \Delta c=\sqrt{A_{n}^{2}+B_{n}^{2}}\\ \varphi_{n}=\frac{1}{n}{\text{tan}}^{-1}(\frac{B_{n}}{A_{n}}) \end{array}$$ (8)

where *φ*_*n*_ is the circumferential position of the maximum deformation of cylinder liner, Δ*c* is the maximum variation of cylinder liner radius from its ideal circular shape, and they are shown in Fig 3. In practice, the variation of Δ*c* is usually very small in the axial direction for the cylinder liner deformation with *n* = 4 [53]. Therefore, a constant value of Δ*c* is assumed in the simulation of cylinder liner deformation in this and other previous studies [22, 25, 49–52]. Furthermore, according to the works of Usman et al. [42], the value of Δ*c* is set to 15 μm in the current study.

Hence, the variation of cylinder liner radius from its inscribed circle Δ*R* can be expressed as follow [25]:

$$\Delta R=\Delta R_{cir}-\Delta R_{\text{min}}$$ (9)

where Δ*R*_min_ is the minimum variation of cylinder liner radius from its ideal circular shape.

It should be noted that the compression ring also elastically deforms to accommodate the variation in cylinder liner radius when the cylinder liner is deformed (i.e., the conformability of compression ring) [48, 50]. The elastic deformation of compression ring caused by the compression ring conformability is expressed as *U*_*n*_, and can be written as [25]:

$$U_{n}=\frac{3\left(F_{t}+F_{bp}\right)r^{2}{\left(2r-a_{r}\right)}^{2}}{E_{1}ba_{r}^{3}{\left(n^{2}-1\right)}^{2}}$$ (10)

where *a*_*r*_ is the radial thickness of compression ring, *r* is the nominal radius of cylinder liner, *E*_1_ is the elasticity modulus of compression ring. *F*_*t*_ is the tension force of compression ring, *F*_*bp*_ is the backpressure force of compression ring, and their expressions are given as follows [27, 50]:

$$F_{t}=\frac{gE_{1}ba_{r}^{3}}{36\pi r^{3}}$$ (11)

$$F_{bp}=2\alpha brp_{g}$$ (12)

where *g* is the end gap of compression ring. *p*_*g*_ is the cylinder pressure, which is change with the crank angle. *α* is the ratio of compression ring backpressure to cylinder pressure. In practice, the determination of the compression ring backpressure is very complicated, and it is closely related to the dynamic behaviors of the compression ring and the gas flow in the ring pack. In this study, according to the works of Yin et al. [54] and Meng et al. [27], a constant value of *α* = 0.7 is assumed to approximately describe the relationship between the compression ring backpressure and cylinder pressure (i.e., the backpressure of compression ring is assumed to be equal to 70% of cylinder pressure) because the compression ring is always in contact with the lower surface of the compression ring groove during the movement [54].

### Oil transport model and pressure boundary condition

**Fig 4:**
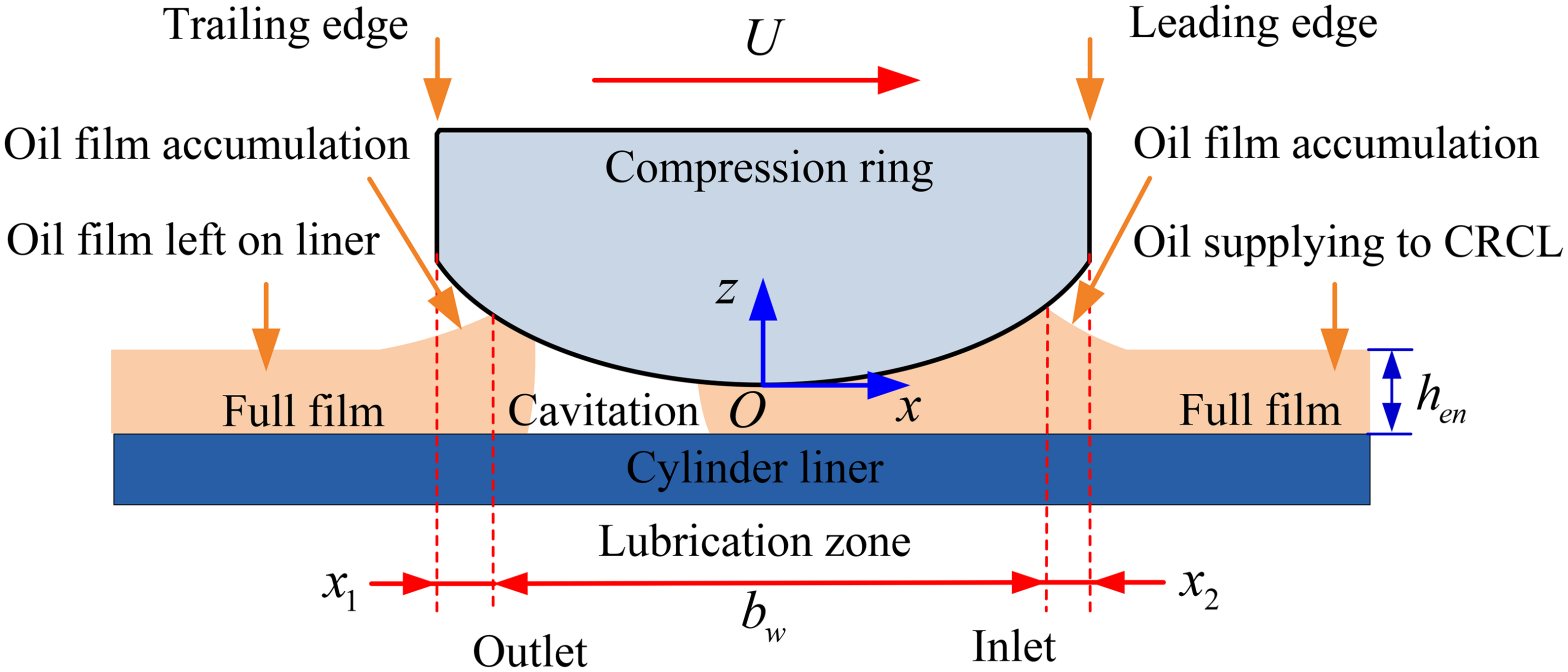
Schematic diagram of the lubricated compression ring-cylinder liner system.

Under engine condition, insufficient oil film is usually supplied to the compression ring-cylinder liner system to provide a hydrodynamic support force because a large amount of oil film has been scraped by the oil control ring [8]. In this case, the starved lubrication and fully flooded lubrication conditions can be encountered simultaneously in an engine cycle because of the time-varying clearance between the compression ring and cylinder liner [48]. In generally, the starved/fully flood lubrication can be distinguished by the axial width of lubrication zone *b*_*w*_, as shown in Fig 4. If *b*_*w*_<*b*, the lubrication condition is starved. However, if *b*_*w*_ = *b*, the lubrication condition is fully flooded.

**Fig 5:**
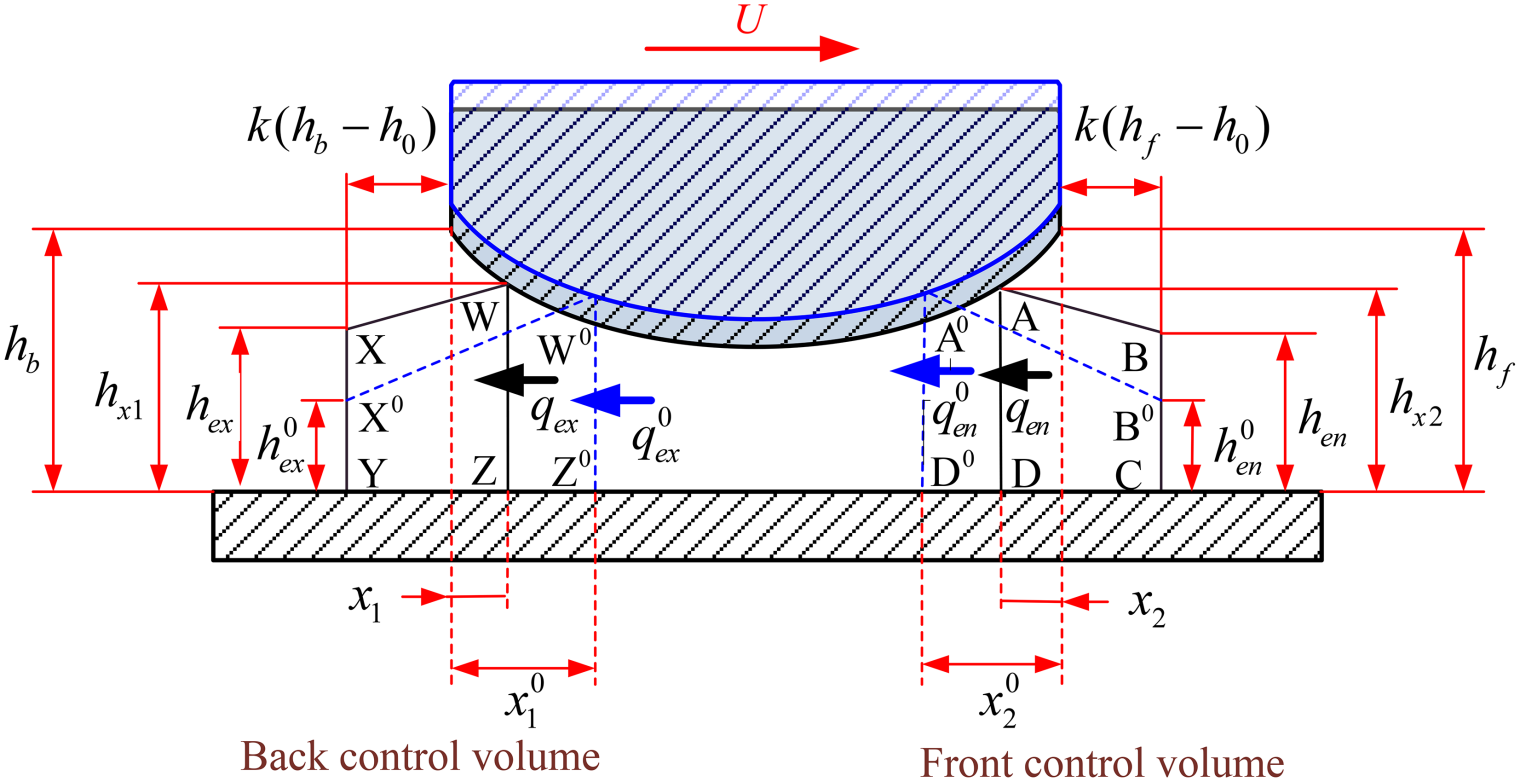
Oil transport model with front and back control volumes at the inlet and outlet of lubricated zone.

Furthermore, it should be pointed out that only a portion of oil film supplying to the compression ring-cylinder liner system will be transported into the lubrication zone [55], and some amount of oil film existing the lubrication zone will be remained at the outlet of the lubrication zone during the operation of the compression ring-cylinder liner system [56]. Hence, some amount of the oil film will be accumulated at the inlet and outlet of the lubrication zone [55, 56]. In order to predicted the transition between fully flooded and starved lubrication conditions in an engine cycle with consideration of the oil film accumulation, an oil transport model [56] with front and back control volumes at the inlet and outlet of the lubricated zone is employed to calculate the width of lubrication zone, as shown in Fig 5.

At the inlet of lubricated zone, the mass balance per unit length in the circumferential direction should be performed for the front volume [56]. This means that the mass variation between the oil film supplying to compression ring-cylinder liner system and the oil film entering the lubrication zone should be equal to the mass variation of oil film in the front control volume.

$$\underset{\begin{array}{l} \;\;\text{Mass}\;\text{of}\;\text{oil}\;\text{film}\\ \text{supplying}\;\text{to}\;\text{CRCL} \end{array}}{\underset{︸}{\frac{Uh_{en}+U^{0}h_{en}^{0}}{2}\rho \Delta t}}-\underset{\begin{array}{l} \text{Mass}\;\text{of}\;\text{oil}\;\text{film}\;\text{entering}\\ \;\;\text{the}\;\text{lubrication}\;\text{zone} \end{array}}{\underset{︸}{\frac{q_{en}+q_{en}^{0}}{2}\rho \Delta t}}=\underset{\begin{array}{l} \;\;\text{Mass}\;\text{variation}\;\text{of}\;\text{oil}\;\text{film}\\ \text{in}\;\text{the}\;\text{front}\;\text{control}\;\text{volume} \end{array}}{\underset{︸}{(S_{ABCD}-S_{A^{0}B^{0}CD^{0}})\rho }}$$ (13)

Similar mass balance should be also performed for the back control volume [56].

$$\underset{\begin{array}{l} \text{Mass}\;\text{of}\;\text{oil}\;\text{film}\;\text{exiting}\\ \;\;\text{the}\;\text{lubrication}\;\text{zone} \end{array}}{\underset{︸}{\frac{q_{ex}+q_{ex}^{0}}{2}\rho \Delta t}}-\underset{\begin{array}{l} \text{Mass}\;\text{of}\;\text{oil}\;\text{film}\;\text{left}\\ \;\;\text{on}\;\text{cylinder}\;\text{liner} \end{array}}{\underset{︸}{\frac{Uh_{ex}+U^{0}h_{ex}^{0}}{2}\rho \Delta t}}=\underset{\begin{array}{l} \text{Mass}\;\text{variationof}\;\text{oil}\;\text{film}\\ \text{in}\;\text{the}\;\text{back}\;\text{control}\;\text{volume} \end{array}}{\underset{︸}{(S_{WXYZ}-S_{W^{0}X^{0}YZ^{0}})\rho }}$$ (14)

where *h*_*ex*_ is the exit height of oil film, Δ*t* is the time interval, *S*_*ABCD*_ and *S*_*WXYZ*_ are the volumes of the front and back control volumes. *q*_*en*_ and *q*_*ex*_ are the oil flow rates at the inlet and outlet of the lubricated zone, and their expressions are defined in reference [27]. *h*_*en*_ is the entry height of oil film, and it means the amount of oil film supplying to the compression ring-cylinder liner system because the relative speed of compression ring is known for each crankshaft angle. The superscript 0 refers to the parameters at the previous time.

The volumes of the front and back control volumes can be given by [56]:

$$S_{ABCD}=\left[k(h_{f}-h_{0})+x_{2}\right](\frac{h_{en}+h_{x2}}{2})$$ (15)

$$S_{WXYZ}=\{\begin{array}{ll} k(h_{b}-h_{0})(\frac{h_{b}+h_{ex}}{2}) & \text{if}\;h_{ex}\geq h_{0}\\ \left[k(h_{x1}-h_{ex})\right](\frac{h_{x1}+h_{ex}}{2}) & \text{if}\;\frac{h_{0}}{10}<h_{ex}<h_{0}\\ \left[k(h_{b}-h_{0})+x_{1}\right]h_{ex}+\frac{k}{2}{\left(h_{x1}-\frac{h_{0}}{10}\right)}^{2} & \text{if}\;h_{ex}<\frac{h_{0}}{10} \end{array}$$ (16)

where *h*_*f*_ and *h*_*b*_ are the clearance heights at the leading and trailing edges of compression ring, *x*_2_ and *x*_1_ are the inlet and outlet widths, *h*_*x*2_ and *h*_*x*1_ are the clearance heights at the inlet and outlet of the lubricated zone. *k* is the aspect ratio, a value of *k* = 100 is adopted according to the previous work [56].

By solving the Eqs (13)–(16), the inlet width *x*_2_ and outlet width *x*_1_ can be obtained for the given oil film entry height *h*_*en*_. On this basis, the width of lubrication zone (*b*_*w*_ = *b*-*x*_1_-*x*_2_) can be calculated, and consequently the transition between the fully flooded lubrication condition and the starved lubrication condition can be predicted for each given amount of oil film supplying to the compression ring-cylinder liner system in an engine cycle.

Moreover, the pressure boundary conditions are necessary for the average Reynolds equation to solve the oil film pressure. If the inlet width *x*_2_ equals to zero, the inlet boundary position of lubrication zone is set at the leading edge of the compression ring (i.e., *x* = *b*/2), and a fully flooded inlet condition is therefore assumed [36]. However, if the inlet width *x*_2_ is greater than zero, the inlet boundary position of lubrication zone is located at *x* = *b*/2-*x*_2_. Thus, the pressure boundary condition at the inlet of the lubrication zone can be written as [44]:

$${p|}_{x=\frac{b}{2}-x_{2}}=\{\begin{array}{l} p_{g}\;\;\;\text{in}\;\text{the}\;\text{compression}\;\text{and}\;\text{exhaust}\;\text{strokes}\\ p_{t}\;\;\;\;\text{in}\;\text{the}\;\text{intake}\;\text{and}\;\text{power}\;\text{strokes} \end{array}$$ (17)

where *p*_*t*_ is the inter-ring pressure, and it can be assumed to be equal to the atmospheric pressure because it is usually slightly higher than the atmospheric pressure in practice.

The pressure boundary condition at the outlet of the lubrication zone should be also paid special attention. Similarly, if the outlet width *x*_1_ equals to zero, the outlet boundary position of lubrication zone is set at the trailing edge of the compression ring (i.e., *x* = -*b*/2). However, if the outlet width *x*_1_ is greater than zero, the outlet boundary position of lubrication zone is located at *x* = -*b*/2+*x*_1_. Thus, the pressure boundary condition at the outlet of the lubrication zone can be written as:

$${p|}_{x=-\frac{b}{2}+x_{1}}=\{\begin{array}{l} p_{t}\;\;\;\text{in}\;\text{the}\;\text{compression}\;\text{and}\;\text{exhaust}\;\text{strokes}\\ p_{g}\;\;\;\text{in}\;\text{the}\;\text{intake}\;\text{and}\;\text{power}\;\text{strokes} \end{array}$$ (18)

### Rheological relationship

The viscosity and density of oil film under operating temperature and pressure affect greatly the simulation results. The variation of oil film viscosity with temperature and pressure can be described by Houpert equation [36].

$$\mu =\mu_{0}\text{exp}\left\{\left(\text{ln}\;\mu_{0}+9.67\right)\left[-1+{\left(1+5.1\times 10^{-9}p\right)}^{z_{0}}{\left(\frac{T-138}{T_{0}-138}\right)}^{-s_{0}}\right]\right\}$$ (19)

with

$$\left\{ \begin{array}{l} z_{0}=\kappa_{vp}/\left[5.1\times 10^{-9}\left(\text{ln}\;\mu_{0}+9.67\right)\right]\\ s_{0}=\kappa_{vt}\left(T_{0}-138\right)/\left(\text{ln}\;\mu_{0}+9.67\right) \end{array}\right.$$ (20)

where *κ*_*vp*_ and *κ*_*vt*_ are the viscosity-pressure and viscosity-temperature coefficients of oil film, *T* is the oil film temperature, *T*_0_ is the reference temperature of oil film, *μ*_0_ is the oil film viscosity under atmospheric pressure when the temperature equals to *T*_0_.

The variation of oil film density with temperature and pressure is also considered in the simulation, and it can be written as [57]:

$$\rho =\rho_{0}\left[1+\frac{0.6\times 10^{-9}p}{1+1.7\times 10^{-9}p}-6.5\times 10^{-4}\left(T-T_{0}\right)\right]$$ (21)

where *ρ*_0_ is the density of oil film under atmospheric pressure when the temperature equals to *T*_0_.

In order to evaluate the rheological properties of oil film, the pressure and temperature distributions of oil film should be known a priori. In generally, the oil film pressure can be obtained by solving the average Reynolds equation, and the oil film temperature can be obtained by the thermal analysis. In this study, a warm engine condition is adopt in the performance simulation of the compression ring-cylinder liner system. According to the work of Gu et al. [19], the thermal effect of oil film has a limited influence on the oil film temperature distribution when the compression ring-cylinder liner system operates under the warm engine condition, and the oil film temperature can be assumed to be equal to the temperature of the cylinder liner. By using the measured cylinder liner temperatures at the piston dead centers, the cylinder liner temperature distribution *T*_*liner*_ can be fitted by [58]:

$$T_{liner}=T_{1}-(T_{1}-T_{2})\sqrt{\frac{m_{1}}{m}}$$ (22)

where *T*_1_ and *T*_2_ are the temperatures of cylinder liner at the top dead center and bottom dead center, *m*_1_ is the distance from top dead center, *m* is the stroke length.

### Asperity contact model

A mixed lubrication analysis is needed for the compression ring-cylinder liner system because it typically operates under the mixed lubrication regime at the piston dead centers (i.e., top and bottom dead centers) [6, 20]. Therefore, the contact behaviors of asperity are unavoidable, and should be taken into consideration.

For the run-in cylinder liner, it is considered to be reasonable to assume that the asperity heights are Gaussian distributed [36, 41]. Then, a statistical model proposed by Greenwood and Tripp can be used to simulate the asperity contact behaviors approximately in the current study because it has the advantages of simplicity and acceptable accuracy [20]. It should be noted that a more accurate simulation on the asperity contact behaviors needs to be further considered for an actual non-Gaussian cylinder liner surface, which can refer to a representative work conducted by Leighton et al [59]. According to the model proposed by Greenwood and Tripp, the contact pressure of asperity *P*_*asp*_ and the contact area of asperity *A*_*c*_ can be expressed by [14]:

$$p_{asp}=\frac{16\sqrt{2}}{15}\pi {\left(\kappa \beta \sigma \right)}^{2}E^{\prime }\sqrt{\frac{\sigma }{\beta }}F_{2.5}(\lambda )$$ (23)

$$A_{c}=\pi^{2}{\left(\kappa \beta \sigma \right)}^{2}AF_{2}(\lambda )$$ (24)

where *E'* is the equivalent elastic modulus of the compression ring-cylinder liner system, and its expression can refer to the work of Meng et al. [27]. *A* is the apparent contact area of asperity, *κ* is the density of asperity, *β* is the mean radius of asperity curvature. *κβσ* is the roughness parameter, *σ/β* is the asperity gradient. In the current study, *σ/β* = 0.001 and *κβσ* = 0.04 are adopted [34]. *λ* is the ratio of the oil film thickness, *F*_2.5_(*λ*) and *F*_2_(*λ*) are the statistic functions of roughness surface, and they can be fitted by [50]:

$$F_{2.5}(\lambda )=-0.0046\lambda^{5}+0.0574\lambda^{4}-0.\text{2958}\lambda^{3}+0.7844\lambda^{2}-1.0776\lambda +0.6167$$ (25)

$$F_{2}(\lambda )=-0.0018\lambda^{5}+0.0281\lambda^{4}-0.1728\lambda^{3}+0.5258\lambda^{2}-0.8043\lambda +0.5003$$ (26)

### Motion equation of compression ring

During the operating of the compression ring-cylinder liner system, the oil film hydrodynamic pressure and the backpressure acting on the compression ring change with the change of crankshaft angles because the velocity and cylinder pressure is time-varying. Therefore, the motion equation of the compression ring and the average Reynolds equation should be solved simultaneously to obtain the time-varying oil film thickness and the frictional characteristics. In practice, the compression ring usually undergoes complex in-plane and out-of-plane dynamic motions. To simplify the tribological analysis, a rigid body motion in the radial direction is assumed/considered in the current study. This assumption/consideration is also adopted in some previous research works [27, 50, 51, 54] to analyze the tribological performance of the compression ring. Generally, the in-plane and out-of-plane dynamic motions of the compression ring will affect the compression ring position in the piston groove and the oil film thickness distribution, and consequently change the performance. Therefore, more accurate analysis can be further conducted by fully considering the in-plane and out-of-plane dynamic motions of the compression ring. The detailed studies on the dynamic motions/behaviors of the compression ring can refer to the works of Baker et al. [60] and Tian et al. [61]. The rigid body motion equation of the compression ring in the radial direction is given as:

$$m_{0}\frac{d^{2}h_{0}}{dt^{2}}=F_{oil}+F_{asp}-F_{bp}-F_{t}$$ (27)

where *m*_0_ is the mass of compression ring. *F*_*oil*_ is the oil film force, *F*_*asp*_ is the contact force of asperity, and they can be calculated by integrating the oil film pressure and asperity contact pressure, respectively. The forces acting on the compression ring are also shown in Fig 1.

Due to the negligible effect of compression ring inertia [12, 13], the inertia item is not considered in the simulation, and consequently the tension force and backpressure force acting on the compression ring are balanced by the oil film force and asperity contact force.

### Performance parameters

The total friction force of the compression ring-cylinder liner system consists of hydrodynamic friction force and asperity friction force. These forces are given as follows [19, 62]:

$$f_{total}=f_{oil}+f_{asp}$$ (28)

$$f_{oil}=\iint_{\Omega }\{-\frac{\mu U}{h}(\varphi_{f}+\varphi_{fs})+\frac{h}{2}\frac{\partial p}{\partial x}\varphi_{fp}\}\text{d}\Omega$$ (29)

$$f_{asp}=\tau_{0}A_{c}+\alpha_{0}F_{asp}$$ (30)

where *f*_*oil*_ is the hydrodynamic friction force, Ω is the area of oil film. *φ*_*f*_, *φ*_*fs*_, and *φ*_*fp*_ are the friction-induced flow factors [36], and their expressions are also given in the Appendix A of S1 File. *f*_*asp*_ is the asperity friction force, and it is a function of the asperity contact area *A*_*c*_ (it can be calculated by using Eq 24) and the asperity contact force *F*_*asp*_. *τ*_0_ is the shear stress constant, and a value of *τ*_0_ = 2.0 MPa is adopted [17, 63]. *α*_0_ is the boundary friction coefficient, and it is closely related to the surface material and topographical properties [17]. The value of *α*_0_ is usually depend on the shear strength of the asperity on the softer of the two counter surfaces (i.e., the cast iron cylinder liner in this case), and can be measured by using an AFM (i.e., atomic force microscope) operating in lateral force mode [15]. A detailed discussion on the AFM measurement can refer to the work of Styles et al. [15]. In this study, a typical empirical value of *α*_0_ = 0.17 for the cast iron cylinder liner is used to simplify the calculation of the asperity friction. In fact, the variation of the value of *α*_0_ is really very small because of the small asperity contact area [25]. Therefore, the typical empirical value of *α*_0_ = 0.17 is considered to be acceptable for the cast iron cylinder liner, and also adopted by some previous works [34, 43, 50].

The power loss of the compression ring-cylinder liner system can be evaluated as [19, 63]:

$$P_{loss}=\left|f_{total}U\right|$$ (31)

Moreover, the friction loss is evaluated by FMEP (friction mean effective pressure) value. The expression of the FMEP can refer to the work of Gu et al. [19].

## Simulation parameters

A typical 4-stroke multi-cylinder gasoline engine at 2000 rpm and full load is considered in this study to analyze the frictional performance of the compression ring-cylinder liner system under the CDA. The gasoline engine is equipped with 6 cylinders, which are arranged in V-shaped double row. In the engine, a steel compression ring and a gray cast iron cylinder liner are used. The deigned geometry parameters and the measured surface topography/mechanical parameters of the compression ring-cylinder liner system are shown in Table 1. Furthermore, the oil 5W40 is used as lubricant, the rheological parameters of the oil 5W40 are summarized in Table 2.

**Table 1:** Engine parameters for the analysis of the compression ring-cylinder liner system.

Parameter	Value
**Crankshaft speed, *n***_***c***_	2000 r/min
**Connecting rod length, *l***	147 mm
**Radius of crankshaft, *R***	47 mm
**Thickness of ring, *a***_***r***_	3.5 mm
**Nominal radius of liner, *r***	42 mm
**End gap of ring, *g***	10 mm
**Surface roughness of ring, *σ***_**1**_	0.4 μm
**Surface roughness of liner, *σ***_**2**_	0.4 μm
**Axial width of ring, *b***	1 mm
**Crown height of ring, *δ***	8 μm
**Elasticity modulus of ring, *E***_**1**_	250 GPa
**Elasticity modulus of liner, *E***_**2**_	120 GPa
**Poisson’s ratio of ring, *υ***_**1**_	0.3
**Poisson’s ratio of liner, *υ***_**2**_	0.3

**Table 2:** Temperatures of cylinder liner at the piston dead centers and the parameters of oil 5W40.

Parameter	Value
**Density, *ρ***	833.8 kg·m^-3^@40 °C
**Viscosity, *μ***	9.59 mPa·s@100 °C
**Thermal conductivity, *k***_***p***_	0.225 W·m^-1^K^-3^@120 °C
**Specific heat capacity, *c***_***p***_	2360 J·kg^-1^K^-3^@120 °C
**Viscosity-pressure coefficient, *κ***_***vp***_	1×10^−8^ m^2^·N^-1^
**Viscosity-temperature coefficient, *κ***_***vt***_	0.026 K^-1^
**Temperature at top dead center, *T***_**1**_	170 °C (Active cylinder) 125 °C (Deactivated cylinder) 160°C (Standard operation)
**Temperature at bottom dead center, *T***_**2**_	142 °C (Active cylinder) 98 °C (Deactivated cylinder) 123°C (Standard operation)

**Fig 6:**
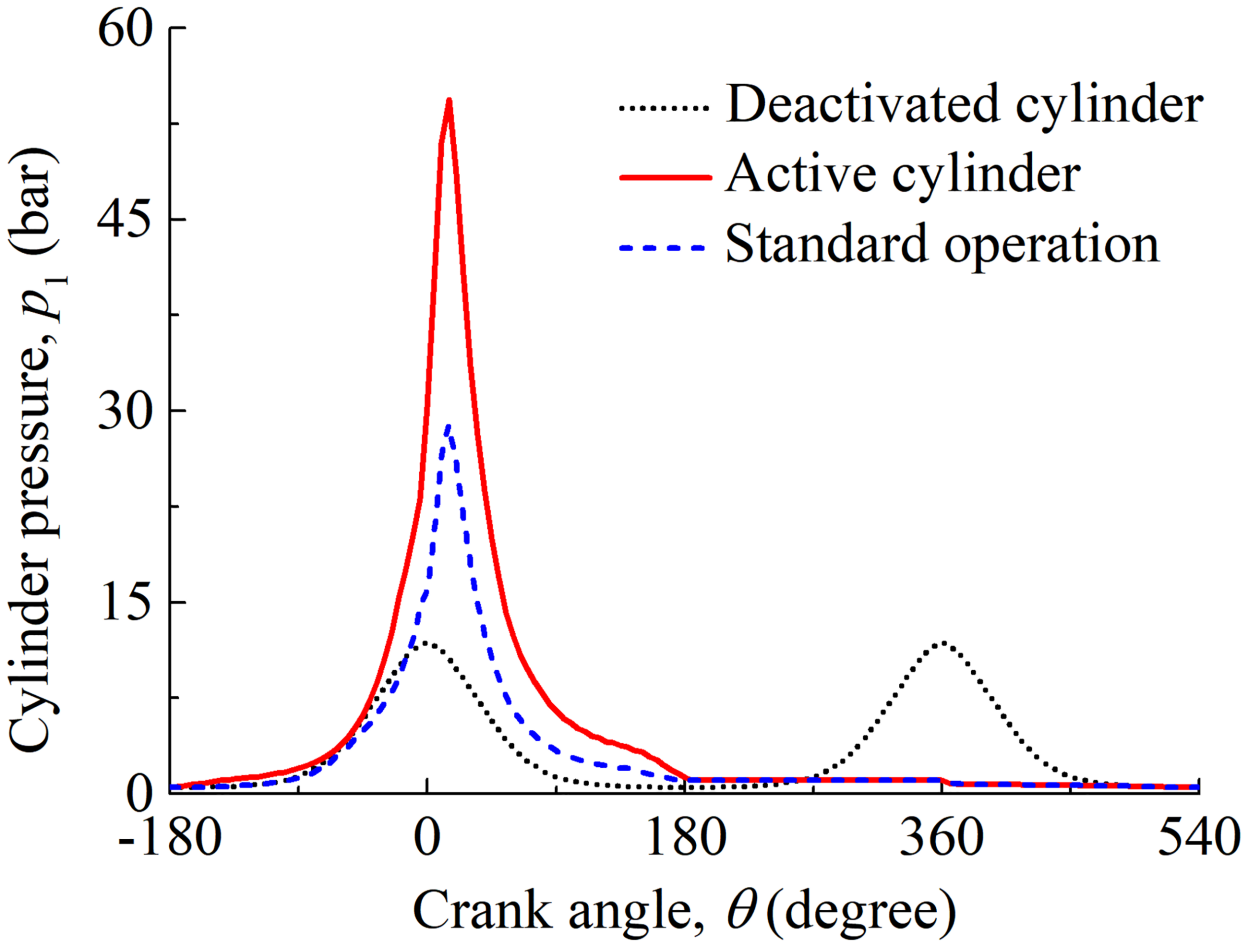
Cylinder pressures for the deactivated and active cylinders under the CDA.

Under the CDA, three of the cylinders are active, and other three cylinders are deactivated (i.e., the ignition is closed, and no power output). The three deactivated cylinders have reduced cylinder pressures and liner temperatures, and the other three active cylinders experience higher cylinder pressures and liner temperatures than that under the standard operation conditions (i.e., the six cylinders are all active) to output desired power. The cylinder pressures for the deactivated cylinder and active cylinder under the CDA are shown in Fig 6. The cylinder liner temperatures at piston dead centers under the CDA are also listed in Table 2.

## Results and discussion

### Validation

**Fig 7:**
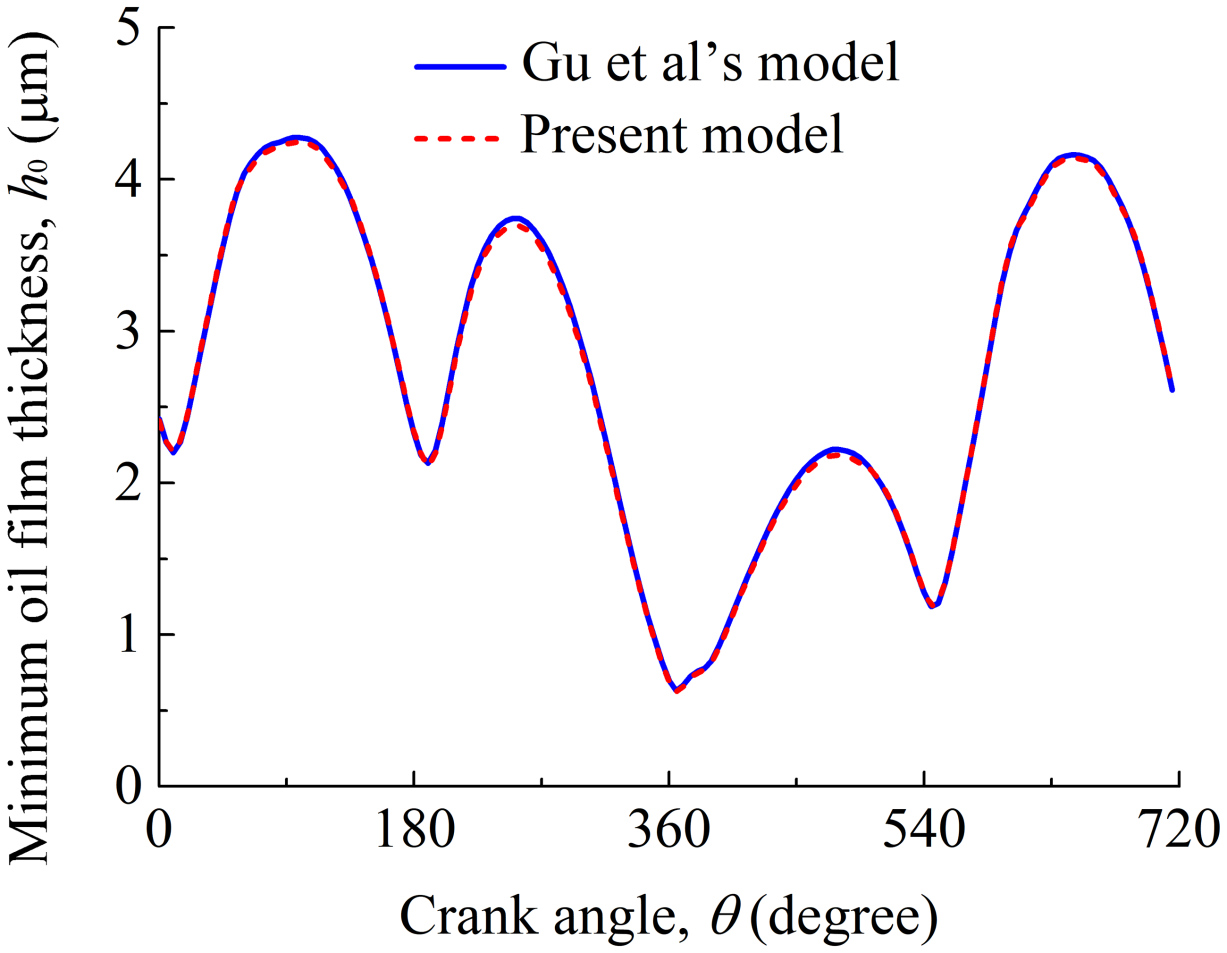
Minimum oil film thicknesses between the compression ring and cylinder liner obtained by Gu et al.’s [64] and our models.

A validation on the present work is needed before investigating the tribological performance of the compression ring-cylinder liner system. The validation has been conducted using Gu et al.’s [64] and our models under the standard operation condition. In the validation, the simulation conditions (i.e., oil film temperature, engine speed, cylinder pressure) are consistent with the work of Gu et al. [64]. The geometry and surface properties of the compression ring and cylinder liner adopted in the validation are also the same as Table 3 in Gu et al.’s work [64]. Fig 7 shows the minimum oil film thicknesses between the compressing ring and the cylinder liner calculated by Gu et al.’s [64] and our models when the crown height of compression ring is 1.0 μm. In Fig 7, the minimum oil film thickness calculated by our model is in good agreement with the results of Gu et al. [64] (i.e., the untextured case in Fig 9(c) in Gu et al.’s [64] work), and the maximum difference of minimum oil film thickness between the two models is not greater than 1%. This result suggests the validation of the present work. Therefore, in what follows, the present model is used to investigate the tribological performance of the compression ring-cylinder liner system under the CDA.

### Influence of oil supply and cylinder liner deformation

Before investigating the tribological performance of the compression ring-cylinder liner system under the CDA, it is also necessary to illustrate the rationality of considering the oil supply and cylinder liner deformation in the analysis. Therefore, three cases (i.e., case 1: with consideration of oil supply and cylinder liner deformation; case 2: with consideration of cylinder liner deformation; case 3: with consideration of oil supply) are considered to investigate the influence of cylinder liner deformation and oil supply on the performance of the compression ring-cylinder liner system under the CDA. It should be noted that the lubrication condition of the compression ring-cylinder liner system is assumed to be fully flooded when the oil supply is not considered (i.e., case 2). When the oil supply is considered (i.e., cases 1 and 3), a small value of oil film entry height *h*_*en*_ = 3 μm is chosen because the oil film supplied to the compression ring-cylinder liner system is usually insufficient.

**Fig 8:**
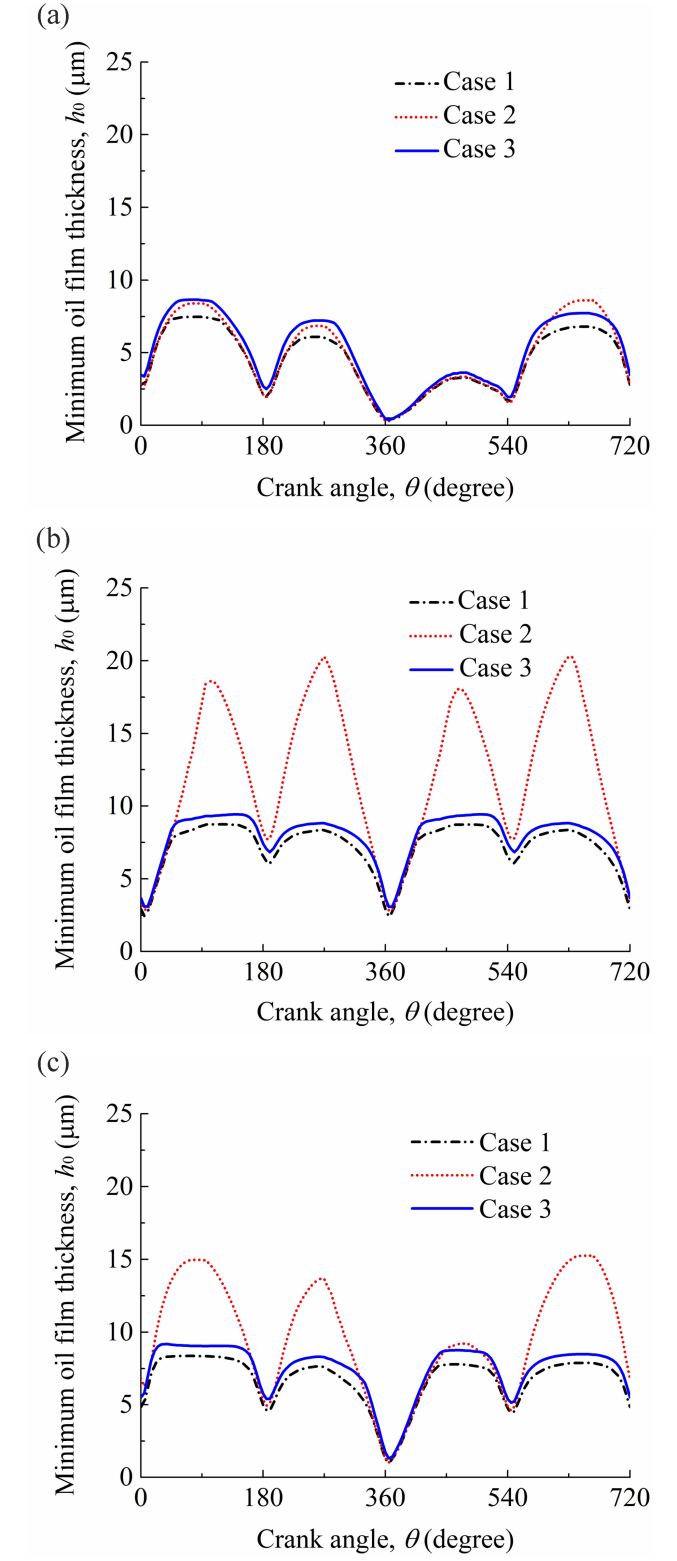
Minimum oil film thicknesses for the three cases. **(a)** active cylinder. **(b)** deactivated cylinder. **(c)** standard operation condition.

Fig 8 shows the minimum oil film thicknesses of the compression ring-cylinder liner system in the active and deactivated cylinders for the three cases. The minimum oil film thicknesses for the compression ring-cylinder liner system under the standard operation condition are also calculated for comparison. As shown in Fig 8, a significant difference in minimum oil film thickness exists among the three cases. Compared with the case 1, higher minimum oil film thicknesses are obtained for the case 2 and case 3, especially when the compression ring operates near the middle of the intake (corresponding to crankshaft angle 0°<*θ*≤180°), compression (corresponding to crankshaft angle 180°<*θ*≤360°), power (corresponding to crankshaft angle 360°<*θ*≤540°), and exhaust (corresponding to crankshaft angle 540°<*θ*≤720°) strokes. This indicates that the oil supply and cylinder liner deformation affect greatly the minimum oil film thickness of the compression ring-cylinder liner system under the CDA, and the minimum oil film thickness is overestimated when the assumptions of circular cylinder liner and fully flooded lubrication condition are adopted in the simulation. Furthermore, from Fig 8(a) and 8(b), it can be also seen that the difference of minimum oil film thickness between the case 2 and case 1 in the deactivated cylinder is larger than that in the active cylinder. It means that the influence of oil supply on the minimum oil film thickness in the deactivated cylinder is much more significant than that in the active cylinder when the compression ring-cylinder liner system operates under the CDA.

**Fig 9:**
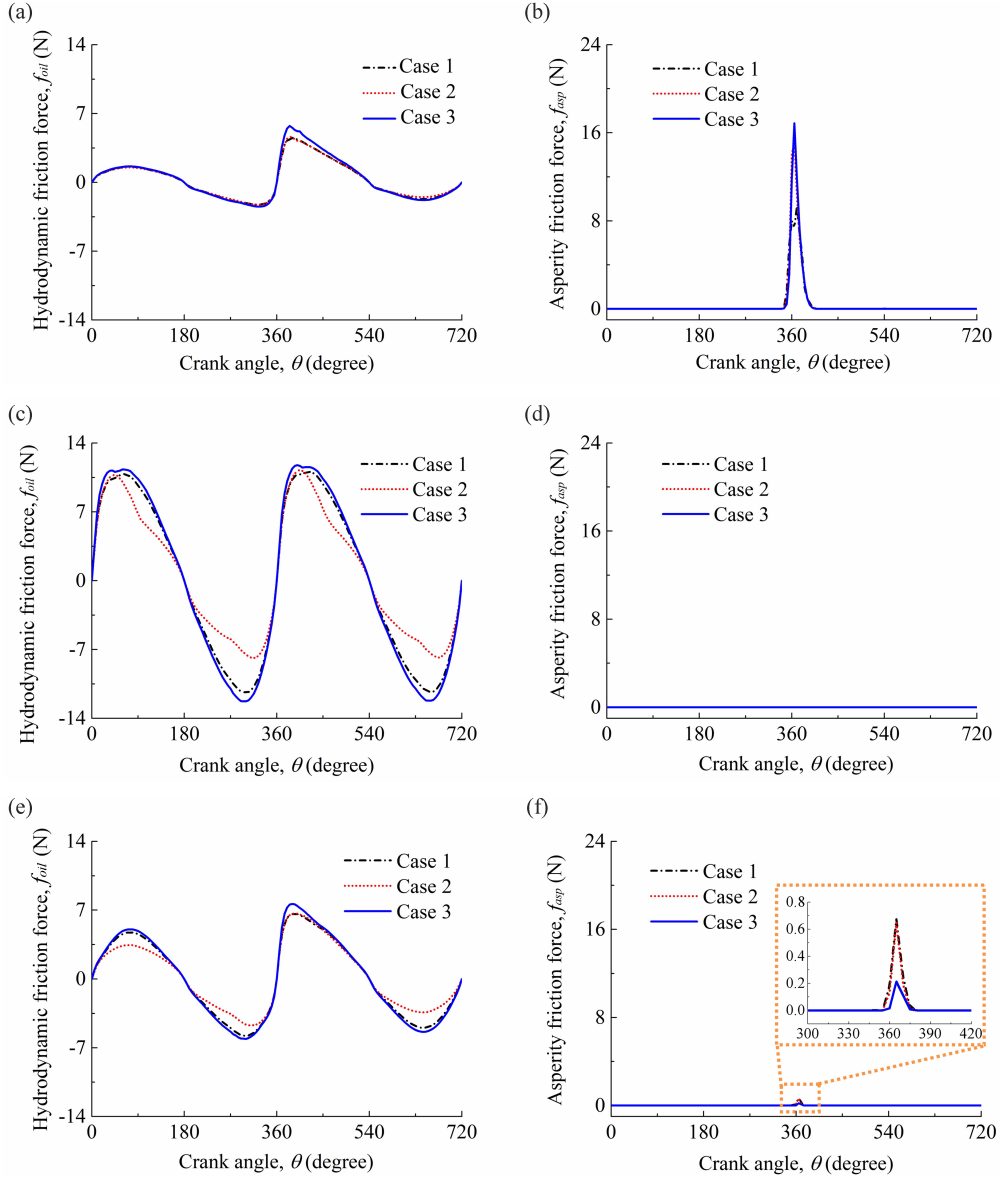
Friction forces for the three cases. **(a)** hydrodynamic frictions in the active cylinder. **(b)** asperity frictions in the active cylinder. **(c)** hydrodynamic frictions in the deactivated cylinder. **(d)** asperity frictions in the deactivated cylinder. **(e)** hydrodynamic frictions under the standard operation condition. **(f)** asperity frictions under the standard operation condition.

Fig 9 shows the friction forces under the three cases for the compression ring-cylinder liner system in the active cylinder, deactivated cylinder, and under the standard operation condition. As shown in Fig 9(a), 9(c) and 9(e), compared with the hydrodynamic friction force under the case 3, smaller hydrodynamic friction force is observed under the case 1 when the compression ring operates near the middle of the strokes. Moreover, by comparing the hydrodynamic friction forces under the case 2 and case 1, a reduced hydrodynamic friction force is observed under the case 2, and the reduction is significant in the middle of the strokes in the deactivated cylinder and under the standard operation condition. However, in the active cylinder, the difference of hydrodynamic friction force between the case 2 and case 1 is negligibly small. Therefore, the cylinder liner deformation affects greatly the hydrodynamic friction force in the active and deactivated cylinders, but the influence of oil supply on the hydrodynamic friction force in the active cylinder is relatively small. Fig 9(b) shows that the asperity friction force of the compression ring-cylinder liner system under the case 1 is smaller than that under the case 2 and case 3 at the top dead center of the power stroke. Hence, the assumptions of circular cylinder liner and fully flooded lubrication overestimate the asperity friction force in the active cylinder. In Fig 9(d) and 9(f), the difference of asperity friction force among the three cases is relatively small, and the asperity friction force of the deactivated cylinder is observed to be equal to zero. This indicates that the cylinder liner deformation and oil supply have limit influence on the asperity friction force in the deactivated cylinder and standard operation condition. Meanwhile, for the compression ring-cylinder liner system in the deactivated cylinder, a pure hydrodynamic lubrication regime exists in the engine cycle.

**Fig 10:**
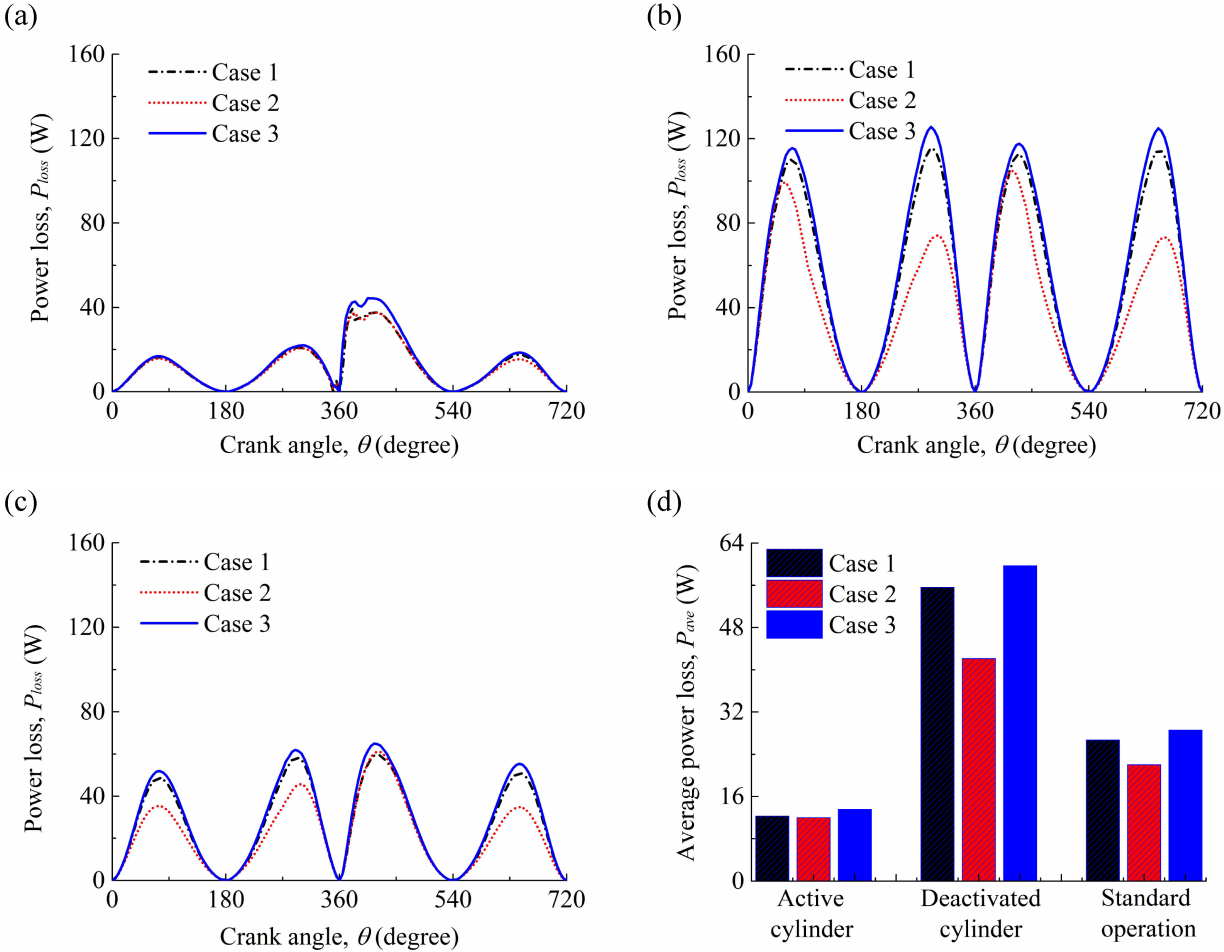
**(a)** Power losses in the active cylinder for the three cases. **(b)** power losses in the deactivated cylinder for the three cases. **(c)** power losses of the compression ring-cylinder liner system under the standard operation condition for the three cases. (d) average power losses of the compression ring-cylinder liner system for the three cases.

The power losses under the three cases for the compression ring-cylinder liner system in the active cylinder, deactivated cylinder, and under the standard operation condition are shown in Fig 10(a), 10(b) and 10(c). Similar with the results of Fig 9(a), 9(c) and 9(e), the power loss under the case 3 is higher than that under the case 1, and the power loss under the case 2 is lower than that under the case 1. This indicates that the assumptions of the ideal circular cylinder liner and fully flooded lubrication will result in an estimation deviation of power loss in the simulation. By comparison between the active cylinder and deactivated cylinder, a significant difference among the three cases is observed in the deactivated cylinder. Therefore, compared with the active cylinder, the influence of the cylinder liner deformation and oil supply on the power loss in the deactivated cylinder is much more significant.

**Fig 11:**
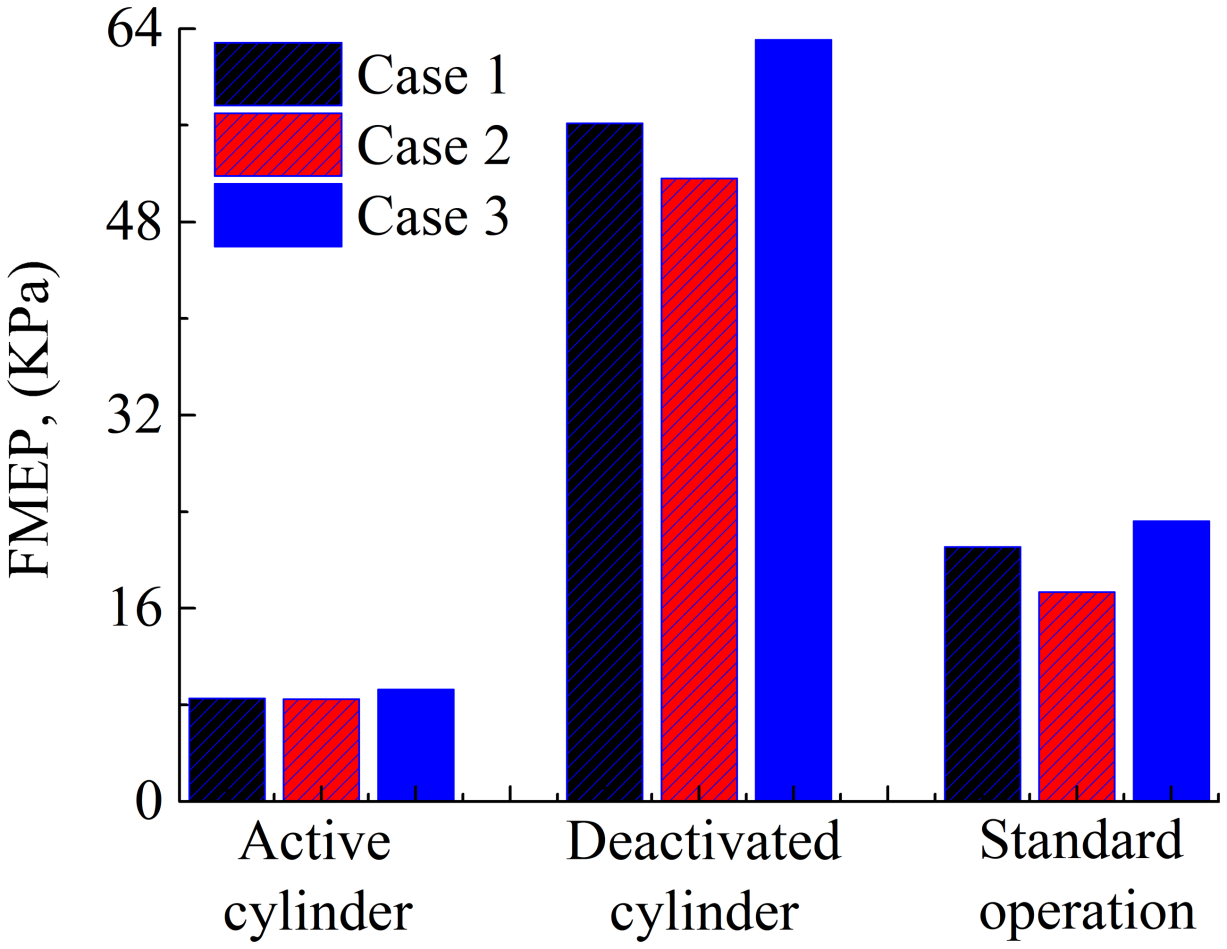
FMEP values of the compression ring-cylinder liner system in the active cylinder, deactivated cylinder, and under standard operation condition for the three cases.

To further clarify the necessity of considering the oil supply and cylinder liner deformation in the analysis, the average power losses and FMEP values (i.e., friction loss) are also calculated for the three cases. The average power losses and FMEP values for the compression ring-cylinder liner system in the active cylinder, deactivated cylinder, and under the standard operation conditions are shown in Figs 10(d) and 11. Compared with the active cylinder and the compression ring-cylinder liner system under the standard operation condition, higher power loss and FMEP value are observed in the deactivated cylinder because of large hydrodynamic friction force. Moreover, the differences of average power loss and FMEP values among the three cases in the deactivated cylinder are also much more significant than that in the active cylinder and standard operation condition. This indicates that the cylinder liner deformation and oil supply affect greatly the average power loss and FMEP value, especially for the compression ring-cylinder liner system in the deactivated cylinder under the CDA.

### Influence of CDA

**Fig 12:**
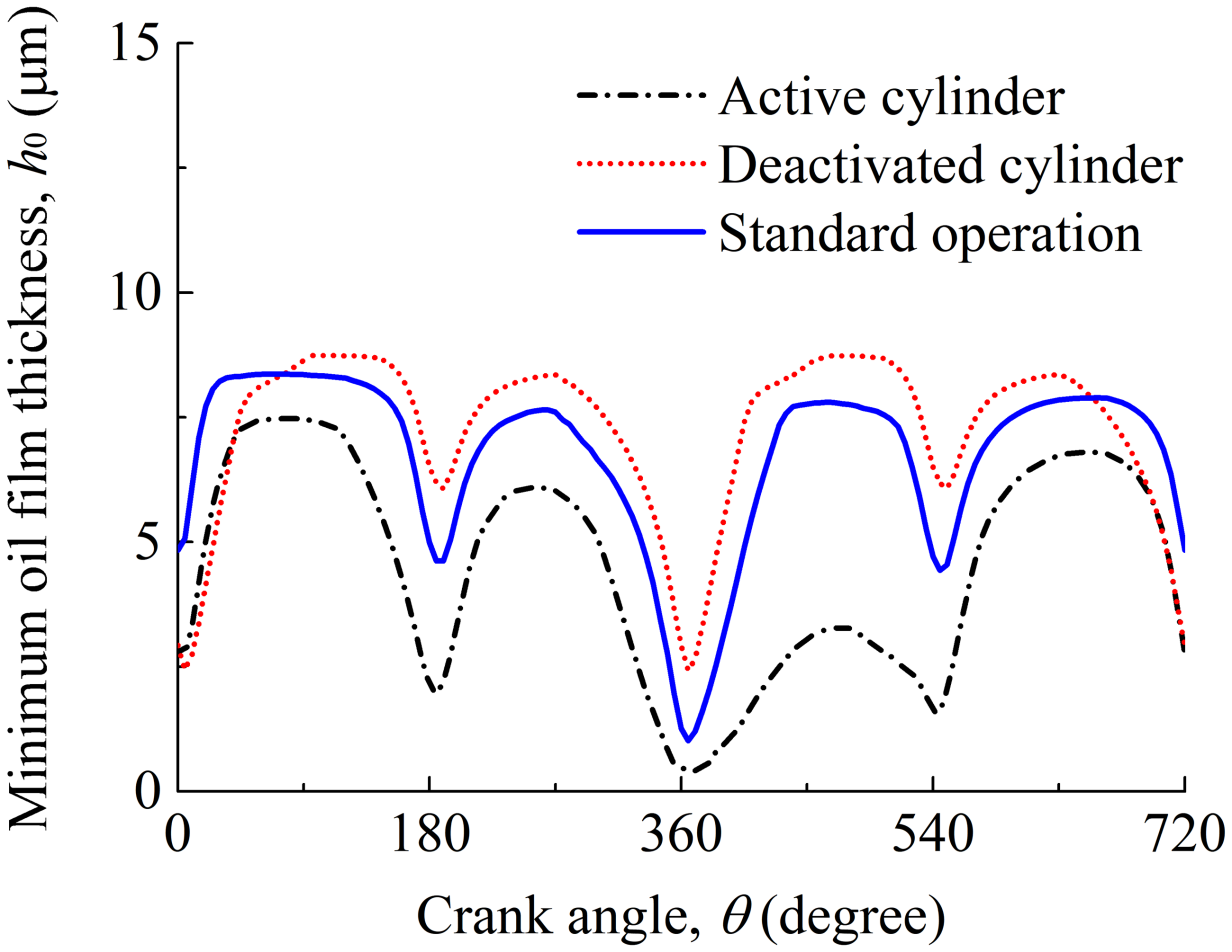
Minimum oil film thicknesses of the compression ring-cylinder liner system in the active cylinder, deactivated cylinder, and under standard operation condition.

With consideration of the oil supply and cylinder liner deformation, the influence of the CDA on the frictional performance of the compression ring-cylinder liner system is also evaluated. Fig 12 shows the minimum oil film thicknesses in the active and deactivated cylinders when the CDA is adopted. For comparison, the minimum oil film thickness under the standard operation condition is also presented. It can be seen that different oil film thicknesses are obtained for the compression ring-cylinder liner system in the active cylinder, deactivated cylinder, and under the standard operation condition. This means that the CDA has a great influence on the minimum oil film thickness of the compression ring-cylinder liner system. In details, compared with the compression ring-cylinder liner system under the standard operation condition, higher minimum oil film thickness is observed for the compression ring-cylinder liner system in the deactivated cylinder in the majority of the engine cycle because of smaller load acting on the compression ring and lower oil film temperature. However, due to the high cylinder pressure and liner temperature in the active cylinder, a reduced minimum oil film thickness is observed, and a significant reduction occurs when the compression ring operates in the power stroke.

**Fig 13:**
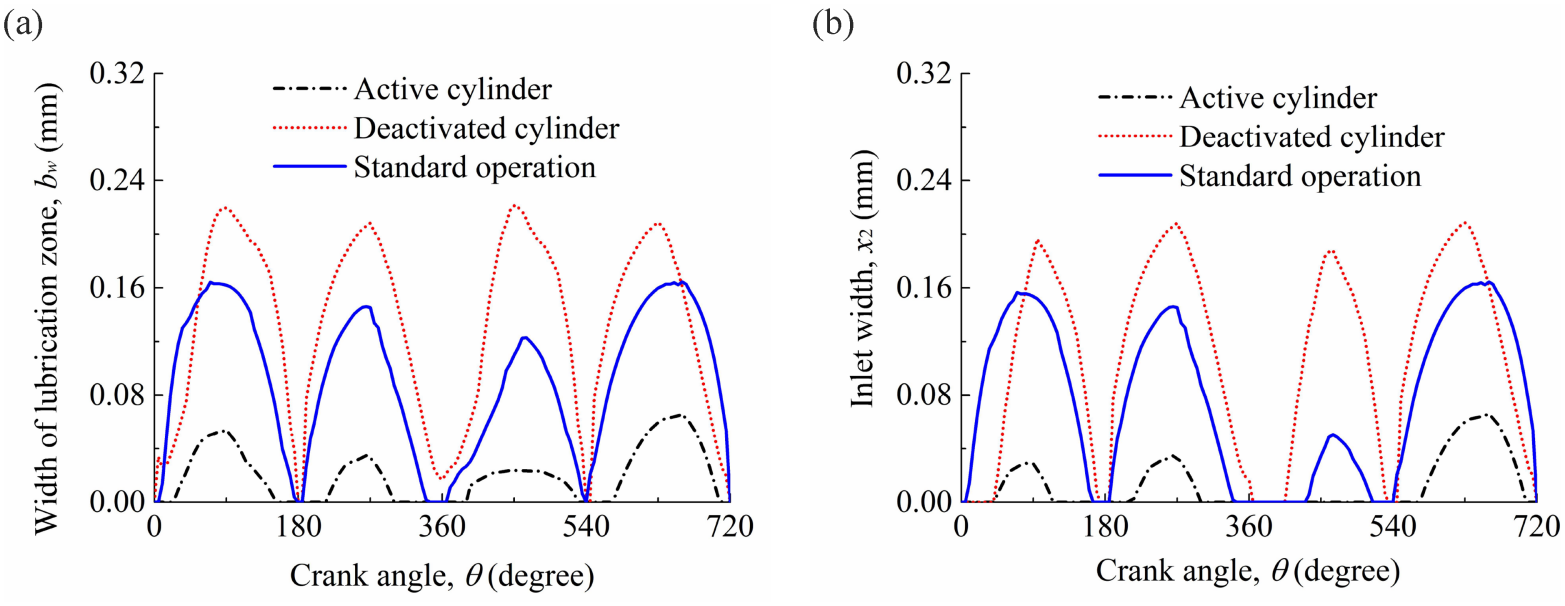
Widths of lubrication zone and inlet widths of compression ring in the active cylinder, deactivated cylinder, and under the standard operation condition.

To predict the transition of lubrication condition under the CDA, Fig 13 shows the widths of lubrication zone and inlet widths of compression ring in the active and deactivated cylinders. In Fig 13, different widths of lubrication zone and inlet widths of compression ring are observed in the active cylinder, deactivated cylinder, and under the standard operation conditions. Compared with the compression ring-cylinder liner system under the standard operation condition, larger width of lubrication zone and inlet width of compression ring are observed for the compression ring-cylinder liner system in the deactivated cylinder, and smaller width of lubrication zone and inlet width of compression ring are observed for the compression ring-cylinder liner system in the active cylinder. Therefore, when the engine changes from the standard operation condition to the CDA, the compression ring in the active cylinder can be covered by oil film more sufficiently in the axial direction, and it consequently experiences a better lubrication condition than the compression ring-cylinder liner system under the standard operation condition. However, in contrast to the results of the compression ring-cylinder liner system in the active cylinder, the starved lubrication condition will be certain to get worse for the compression ring-cylinder liner system in the deactivated cylinder. Furthermore, from Fig 13(a), it can be also seen that the width of lubrication zone reduces to zero in the active cylinder when the compression ring operates near the dead centers (*θ* = 0°, 180°, 360°, 540°, and 720°). This indicates that the compression ring-cylinder liner system in the active cylinder is usually fully flooded at the piston dead centers, and changing from the fully flooded lubrication to starved lubrication when the compression ring reaches near the middle of the strokes.

**Fig 14:**
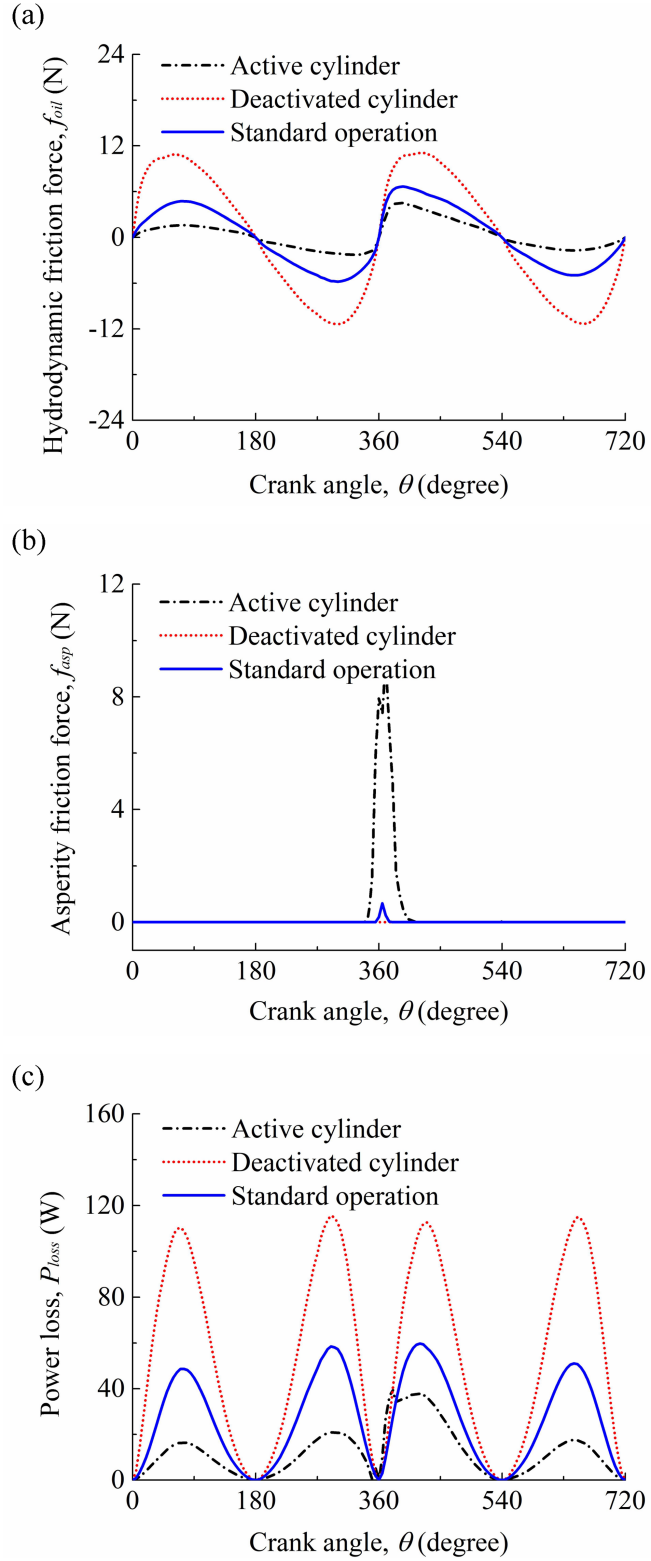
**(a)** Hydrodynamic frictions for the compression ring-cylinder liner system in the active cylinder, deactivated cylinder, and under the standard operation condition. **(b)** asperity frictions for the compression ring-cylinder liner system in the active cylinder, deactivated cylinder, and under the standard operation condition. **(c)** power losses for the compression ring-cylinder liner system in the active cylinder, deactivated cylinder, and under the standard operation condition.

Fig 14(a) and 14(b) show the hydrodynamic friction forces and asperity friction forces for the compression ring-cylinder liner system in the active cylinder, deactivated cylinder, and under the standard operation condition. In Fig 14(a) and 14(b), the compression rings in the active cylinder, deactivated cylinder, and under the standard operation condition have different hydrodynamic friction forces and asperity friction forces, and the difference of hydrodynamic friction force is significant when the compression ring reaches near the middle of the strokes. Compared with the compression ring-cylinder liner system under the standard operation condition, lower hydrodynamic friction force and higher asperity friction force are observed in the active cylinder because of lower viscosity of oil film. Meanwhile, it can be seen that the hydrodynamic friction force in the deactivated cylinder is significantly increased when the engine changes from the standard operation condition to the CDA, especially in the middle of the strokes. Fig 14(c) presents the power losses of the compression ring-cylinder liner system. Compared with the power loss of the compression ring-cylinder liner system in the standard operation condition, a reduced power loss is observed for the compression ring-cylinder liner system in the active cylinder, and an increased power loss is observed in the deactivated cylinder.

To clearly show the changes of the power loss and friction loss after adopting the CDA, the average power losses and FMEP values in the active cylinder, deactivated cylinder, and under the standard operation condition are also summarized in Table 3. Compared with the compression ring-cylinder liner system under the standard operation condition, the average power loss and FMEP value are reduced about 54.0% and 59.4% when the compression ring operates in the active cylinder, and increased about 108.5% and 166.4% when the compression ring operates in the deactivated cylinder. For the 6-cylinder gasoline engine under the standard operation condition (not under the CDA), the total average power loss of the six compression rings is 160.08 W, and the total FMEP value of the six compression rings is 126.48 KPa. However, for the 6-cylinder gasoline engine under the CDA (three cylinders are active, and other three cylinders are deactivated), the total average power loss of the six compression rings is 203.76 W, and the total FMEP value of the six compression rings is 194.16 KPa. Therefore, 27.29% increase in the total average power loss and 53.51% increase in the total FMEP value can be observed for the engine under the CDA.

**Table 3:** Average power losses and FMEP values for the compression ring-cylinder liner system in the active cylinder, deactivated cylinder, and under the standard operation condition.

Parameters	Value		
	Active cylinder	Deactivated cylinder	Standard operation
**Average power loss, *P***_***ave***_	12.28 W	55.64 W	26.68 W
**FMEP value**	8.56 KPa	56.16 KPa	21.08 KPa

## Conclusions

By considering the cylinder liner deformation and oil supply, the tribological performance of the compression ring-cylinder liner system under the CDA is analyzed in this study. A mixed lubrication model and an oil transport model coupled with JFO conservative cavitation algorithm are proposed to evaluate the frictional properties, and to predict the transition between the fully flooded lubrication and starved lubrication. The related conclusions are made as follows:

Under the CDA, the cylinder liner deformation and oil supply show great influence on the frictional performance of the compression ring-cylinder liner system. The power loss and FMEP value are overestimated when the cylinder liner is assumed to be circular, and underestimated when the oil supply is not considered. Therefore, the cylinder liner deformation and oil supply should be considered in the analysis.

With consideration of cylinder liner deformation and oil supply, the CDA has a significant effect on the tribological behaviors of the compression ring-cylinder liner system. In particular, compared with the engine under the standard operation condition, the overall power loss and FMEP value are increased about 27.29% and 53.51% for the engine under the CDA. Certainly, the increased proportion mainly depends on the working conditions of the compression ring-cylinder liner system and the engine type. An increase in fuel and power loss under the CDA is also shown by Morris et al. [36] and Bewsher et al. [34] for an ideal circular cylinder liner under fully flooded lubrication condition. The results of this study demonstrate that it is necessary to consider the performance deterioration of the compression ring-cylinder liner system when the CDA is adopted in the gasoline engine.

The tribological performance deterioration of compression ring-cylinder liner system under the CDA is mainly caused by the changes of the oil film temperature and cylinder pressure. Therefore, in future developments of the CDA technology, the selections of suitable ring/liner material and surface treatment technology (e.g., coating) can be expected to control the oil film temperature and consequently improve the tribological performance under the CDA by changing the heat conduction speed. Furthermore, texturing micro dimples/grooves on the surfaces of compression ring and cylinder liner can also relieve the tribological performance deterioration caused by the CDA to a certain extent.

It should be noted that an isothermal assumption is adopted in this study. In practice, the friction heat will be conducted to the compression ring and cylinder liner, and increases the oil film temperature simultaneously. The increased oil film temperature will alter the oil film viscosity and density, and consequently affect the tribological performance. Therefore, a thermal analysis is required in the tribological analysis of the compression ring-cylinder liner system. Moreover, another assumption made in the current analysis is that the compression ring only undergoes rigid body radial motion. The complex in-plane and out-of-plane dynamic motions of the compression ring also need to be further considered. Therefore, the analysis with consideration of compression ring in-plane and out-of-plane dynamic motions under thermal condition is the future direction of the current research.

## Supporting information

S1 File Supplementary appendices. S1 File include appendix A and B. Appendix A describes the flow factors and contact factor used in this study, and appendix B shows the nomenclature, Greek symbols, abbreviations, and keywords.
(DOCX)
