# Supplementary material for: Evaluation on the tribological performance of ring/liner system under cylinder deactivation with consideration of cylinder liner deformation and oil supply
Source: PLoS One. 2018 Sep 17;13(9):e0204179. doi: 10.1371/journal.pone.0204179 (PMC6141092; doi:10.1371/journal.pone.0204179)
Supplement: S1 File — include appendix A and B. Appendix A describes the flow factors and contact factor used in this study, and appendix B shows the nomenclature, Greek symbols, abbreviations, and keywords. (DOCX) [file pone.0204179.s001.docx]

**Appendix A: The flow factors and contact factor used in this study.**

The pressure flow factors *ϕ_x_* and *ϕ_y_* can be estimated by the expressions proposed by Patir and Cheng [38], and the expressions are given as follows:

 ( A. 1)

 ( A. 2)

where *γ* is the ratio of the roughness correlation lengths in the *x* and *y* directions. The surface roughness is isotropic when *γ* equals to one*.*

The shear flow factor *ϕ_s_* is given by [39]:

 (A. 3)

where *V*_1_ and *V*_2_ are the variance ratios, and they can be expressed as:

$\left\{\begin{matrix} V_{1}=\sigma_{1}^{2}/\sigma^{2} \\ V_{2}=\sigma_{2}^{2}/\sigma^{2} \end{matrix}\right.$ ( A. 4)

The contact factor (*ϕ_c_*) can be estimated by [40]:

 ( A. 5)

The friction-induced flow factors *φ_f_*, *φ_fs_*, and *φ_fp_* are given by the following relationships [36]:

 ( A. 6)

 ( A. 7)

 ( A. 8)

with

$\xi=\lambda/3$ ( A. 9)

$N_{1}=\xi\left\{\xi\left[132+\xi(M_{1}+345)\right]\right\}-55$ ( A. 10)

$M_{1}=\xi\left\{\xi\left[\xi(60+147\xi)-405\right]-160\right\}$ ( A. 11)

**Appendix B: Nomenclature, Greek symbols, abbreviations, and keywords**

**Nomenclature**

| *a_r_* | radial thickness of compression ring |
| --- | --- |
| *A* | apparent contact area of asperity |
| *A_c_* | asperity contact area |
| *A_n_* | Fourier coefficient for the cosine function with order *n* |
| *b* | axial width of compression ring |
| *b_w_* | width of lubrication zone |
| *B_n_* | Fourier coefficient for the sine function with order *n* |
| *c_p_* | specific heat capacity of oil film |
| Δ*c* | maximum variation of cylinder liner radius from its ideal circular shape |
| *E*_1_ | elastic modulus of compression ring |
| *E*_2_ | elastic modulus of cylinder liner |
| *E'* | equivalent elastic modulus of the ring/liner system |
| *f_total_* | total friction force |
| *F_asp_* | asperity contact force |
| *f_asp_* | asperity friction force |
| *F_bp_* | backpressure force of compression ring |
| *F_t_* | tension force of compression ring |
| *f_oil_* | hydrodynamic friction force |
| *F_oil_* | oil film force |
| *F*_2.5_(*λ*), *F*_2_(*λ*) | statistic functions of roughness surface |
| *g* | end gap of compression ring |
| *h* | oil film thickness |
| *h*_0_ | minimum oil film thickness |
| *h_b_* | clearance height at the trailing edge of compression ring |
| *h_en_* | oil film entry height |
| *h_ex_* | oil film exit height |
| *h_f_* | clearance height at the leading edge of compression ring |
| *h_x_*_2_ | clearance height at the inlet of oil lubricated zone |
| *h_x_*_1_ | clearance height at the outlet of oil lubricated zone |
| *h_ring_* | gap caused by face profile of compression ring |
| *h_liner_* | gap caused by cylinder liner deformation and compression ring conformability |
| *k* | aspect ratio |
| *k_p_* | thermal conductivity of oil film |
| *l* | connecting rod length |
| *m* | stroke length |
| *m*_0_ | mass of compression ring |
| *m*_1_ | distance from top dead center |
| *n* | order of Fourier series |
| *N* | maximum order of Fourier series |
| *n_c_* | crankshaft speed |
| *p* | oil film pressure |
| *p_asp_* | asperity contact pressure |
| *p_c_* | cavitation pressure |
| *p_g_* | cylinder pressure |
| *p_t_* | inter-ring pressure |
| *P_loss_* | power loss |
| *q_en_* | inlet oil flow rate |
| *q_ex_* | outlet oil flow rate |
| *r* | nominal radius of cylinder liner |
| *R* | radius of crankshaft |
| Δ*R* | variation of cylinder liner radius from its inscribed circle |
| Δ*R_cir_* | variation of cylinder liner radius from its ideal circular shape |
| Δ*R*_min_ | minimum variation of cylinder liner radius from its ideal circular shape |
| *t* | time |
| Δ*t* | time interval |
| *T* | Temperature of oil film |
| *T*_0_ | oil temperature under atmospheric pressure |
| *T*_1_ | temperature of cylinder liner at top dead center |
| *T*_2_ | temperature of cylinder liner at bottom dead center |
| *T_liner_* | temperature of cylinder liner |
| *U* | relative velocity of compression ring |
| *U_n_* | elastic deformation of compression ring |
| *S_ABCD_, S_WXYZ_* | volumes of front and back control volumes at the current time |
| *S_A_*^0^*_B_*^0^*_CD_*^0^*, S_W_*^0^*_X_*^0^*_YZ_*^0^ | volumes of front and back control volumes at the previous time |
| *x* | axial coordinate in global coordinate system |
| *x*_2_, *x*_1_ | inlet and outlet widths |
| *y* | circumferential coordinate in global coordinate system |

**Greek Symbol**

| *α* | ratio of compression ring backpressure to cylinder pressure |
| --- | --- |
| *α*_0_ | asperity friction coefficient |
| *β* | mean radius of asperity curvature |
| *γ* | ratio of the roughness correlation lengths |
| *δ* | crown height of compression ring |
| *θ* | crankshaft angle |
| *θ_c_* | saturation of oil film |
| *κ* | density of asperity |
| *κ_vp_* | viscosity-pressure coefficient of oil film |
| *κ_vt_* | viscosity-temperature coefficient of oil film |
| *λ* | ratio of oil film thickness |
| *μ* | oil film viscosity |
| *μ*_0_ | oil film viscosity under atmospheric pressure |
| *υ*_1_ | Poisson’s ratio of compression ring |
| *υ*_2_ | Poisson’s ratio of cylinder liner |
| *ρ* | oil film density |
| *ρ*_0_ | oil film density under atmospheric pressure |
| *σ* | comprehensive surface roughness of ring and liner |
| *σ*_1_ | surface roughness of compression ring |
| *σ*_2_ | surface roughness of cylinder liner |
| *τ*_0_ | shear stress constant |
| *φ* | circumferential position |
| *φ_n_* | circumferential position of maximum deformation of cylinder liner |
| *φ_f_*, *φ_fs_*, *φ_fp_* | friction-induced flow factors |
| *ϕ_s_*, *ϕ_c_* | shear flow and contact factors |
| *ϕ_x_*, *ϕ_y_* | pressure flow factors |
| Ω | area of oil film |

**Abbreviations**

| AFM | atomic force microscope |
| --- | --- |
| CDA | cylinder deactivation |
| FMEP | friction mean effective pressure |
| JFO | Jacobson, Floberg, and Olsson |
| VVA | variable valve actuation |

**Keywords:** gasoline engine under cylinder deactivation, deformed cylinder liner, oil supply, power loss, friction mean effective pressure (FMEP).
